# CLICK-chemoproteomics and molecular dynamics simulation reveals pregnenolone targets and their binding conformations in Th2 cells

**DOI:** 10.3389/fimmu.2023.1229703

**Published:** 2023-10-31

**Authors:** Sougata Roy, Sudeep Roy, Bidesh Mahata, Jhuma Pramanik, Marco L. Hennrich, Anne-Claude Gavin, Sarah A. Teichmann

**Affiliations:** ^1^ Department of Biology, Ashoka University, Rajiv Gandhi Education City, Sonipat, Haryana, India; ^2^ Department of Biomedical Engineering, Faculty of Electrical Engineering and Communication, Brno University of Technology, Brno, Czechia; ^3^ Division of Immunology, Department of Pathology, University of Cambridge, Cambridge, United Kingdom; ^4^ Structural and Computational Biology Unit, European Molecular Biology Laboratory, EMBL, Heidelberg, Germany; ^5^ Cellzome, a GlaxoSmithKline (GSK) company, Genomic Sciences, Pharma R&D, Heidelberg, Germany; ^6^ Department for Cell Physiology and Metabolism, Centre Medical Universitaire, University of Geneva, Geneva, Switzerland; ^7^ Diabetes Center, Faculty of Medicine, University of Geneva, Geneva, Switzerland; ^8^ Cellular Genetics, Wellcome Sanger Institute, Cambridge, United Kingdom; ^9^ Theory of Condensed Matter, Cavendish Laboratory, Cambridge, United Kingdom

**Keywords:** pregnenolone, lymphosteroid, chemoproteomics, TH2, click chemistry

## Abstract

Pregnenolone (P5) is synthesized as the first bioactive steroid in the mitochondria from cholesterol. Clusters of differentiation 4 (CD4+) and Clusters of differentiation 8 (CD8+) immune cells synthesize P5 *de novo*; P5, in turn, play important role in immune homeostasis and regulation. However, P5’s biochemical mode of action in immune cells is still emerging. We envisage that revealing the complete spectrum of P5 target proteins in immune cells would have multifold applications, not only in basic understanding of steroids biochemistry in immune cells but also in developing new therapeutic applications. We employed a CLICK-enabled probe to capture P5-binding proteins in live T helper cell type 2 (Th2) cells. Subsequently, using high-throughput quantitative proteomics, we identified the P5 interactome in CD4+ Th2 cells. Our study revealed P5’s mode of action in CD4+ immune cells. We identified novel proteins from mitochondrial and endoplasmic reticulum membranes to be the primary mediators of P5’s biochemistry in CD4+ and to concur with our earlier finding in CD8+ immune cells. Applying advanced computational algorithms and molecular simulations, we were able to generate near-native maps of P5–protein key molecular interactions. We showed bonds and interactions between key amino acids and P5, which revealed the importance of ionic bond, hydrophobic interactions, and water channels. We point out that our results can lead to designing of novel molecular therapeutics strategies.

## Introduction

Pregnenolone (P5), the first bioactive steroid hormone of the steroid biosynthesis pathway, is synthesized in mitochondria from cholesterol. P5 is the progenitor of all glucocorticoids, mineralocorticoids, androgens, estrogens, and progesterone (1, 2). Adrenal, gonads, and placenta synthesize P5 (3). Moreover, several steroidogenic cells have been reported to synthesize P5 (4). The role of P5 in the nervous system has been widely acknowledged (5, 6). P5 enhances synapse and myelinization and induces growth of neurites (7). P5 and its derivatives boost cognition and memory (6) and also demonstrate therapeutic prospects in schizophrenia (8). P5 in prostate cancer (9, 10) and melanoma promotes tumor growth, whereas, in glioma, it restricts tumor growth (11). P5 mediates anti-inflammatory properties by activating degradation of proteins in the Toll-like receptor signaling pathway (12). Many cytoskeletal proteins are P5 receptors and regulate microtubule dynamics, cell migration, and mitotic cell division (13, 14).

The discovery of P5 as a lymphosteroid was reported recently by confirming its synthesis in lymphocytes and immune cells that infiltrate tumor (15, 16). P5 synthesis in immune cells is a newly emerging domain that reveals the functional diversity of P5 across different cell types. In type 2 CD8+ cells, local P5 synthesis drives its differentiation program (16). CD4+ T cells are key mediators of immune responses against a plethora of infections (17) and cancers (18). Th2 lymphocytes are a primary subset of T cells that play key defensive roles to counter bacterial and helminth (19) infections mounting adaptive immune responses (20, 21). Th2 cells can synthesize P5, which, in turn, regulates their proliferation and class switching activity of B cells. In Th2 cells, P5 seems to play a crucial role in restoring immune homeostasis (15).

Understanding P5’s regulatory action on Th2 immune cells becomes a topic of utmost importance. How does P5 regulate Th2 cellular biochemistry? To address this in our earlier study, we used an interdisciplinary approach combining synthetic chemistry with high-throughput mass spectrometry. We designed and manufactured a synthetic and photoactivatable P5 analog (22). We captured P5 interactome in the cancer and CD8+ immune cells, the first proteome-wide P5 interactome map in any living cell. We identified 62 prospective target proteins of P5. The functional mapping of target proteins revealed a P5’s non-genomic mode of action. However, our analyses showed that distinct pathways are targeted by P5 in cancer and CD8+ cells (22). Th2 cell proliferation is inhibited by P5; however, the mechanistic understanding is missing.

Details of protein–ligand molecular interaction provides deep insight into the underlying mechanisms of bioregulation (23). It also imparts novel structural information required for designing and development of novel drug molecules (24). Molecular docking (MD) algorithms predict the mode and energy of binding in a ligand–target protein interaction. Molecular dynamics simulations not only improve MD prediction scores but also provide an atomic level resolution of the structure and dynamics of protein–ligand interactions. Over the last two decades, more than 60 different docking tools and programs have been developed for both academic and commercial use such as DOCK (25), AutoDock (26), FlexX (27), Surflex (28), GOLD (29), ICM (30), Glide (31), Cdocker, LigandFit (25), MCDock, FRED (32), MOE-Dock (33), LeDock (34), AutoDock Vina (35), rDock (36), and UCSF Dock (37).

However, the absolute energies associated with the intermolecular interaction are not estimated with satisfactory accuracy by the current algorithms. The major issues of solvent effects, entropic effects, and receptor flexibility still need to be handled with special attention. As of now, some methods like MOE-Dock, GOLD, Glide, FlexX, and Surflex that deal with side chain flexibility have been proven effective and adequate in most of the cases. The realistic interactions between small molecules and receptors still rely on experimental technology. Computational techniques can assist in suggesting modifications to existing structures for obtaining desirable properties and functions. In MOE2022.02, the Protein Design application permits unlimited simultaneous residue mutations of target structures and then calculates the properties of the resultant mutant with the purpose of examining how mutations of a protein modulate its protein properties.

By combining MD and molecular dynamics simulation, near-native binding conformations can be achieved. P5 target protein–binding mode, its structure, and the underlying key interactions are unknown.

In this study, we have used the CLICK-enabled cell-permeable P5 probe (22) in living Th2 cells. In conjunction with high-throughput quantitative proteomics, we identified 11 “P5-binding” target proteins in Th2 immune cells that are localized in the endoplasmic reticulum and mitochondria. Using an *in silico* molecular simulation dynamics, we present structural insight of the molecular interaction between P5 and its key targets. The identified P5 targets belong to some key pathways such as sterol biosynthesis, transport, protein processing, and mitochondrial organization that clearly endorse the non-genomic role of P5’s in immune homeostasis. Most importantly, our results suggest that P5 activity in immune cells is mediated via mitochondria and Endoplasmic reticulum (ER). We envisage that our study will not only provide a mechanistic understanding of the pregnenolone biochemistry in immune cells but also reveal the particulars of the binding efficiency and structural details of P5–target protein interaction.

## Results

### P5-C captures pregnenolone binding proteins from murine Th2 cells

**Figure 1:**
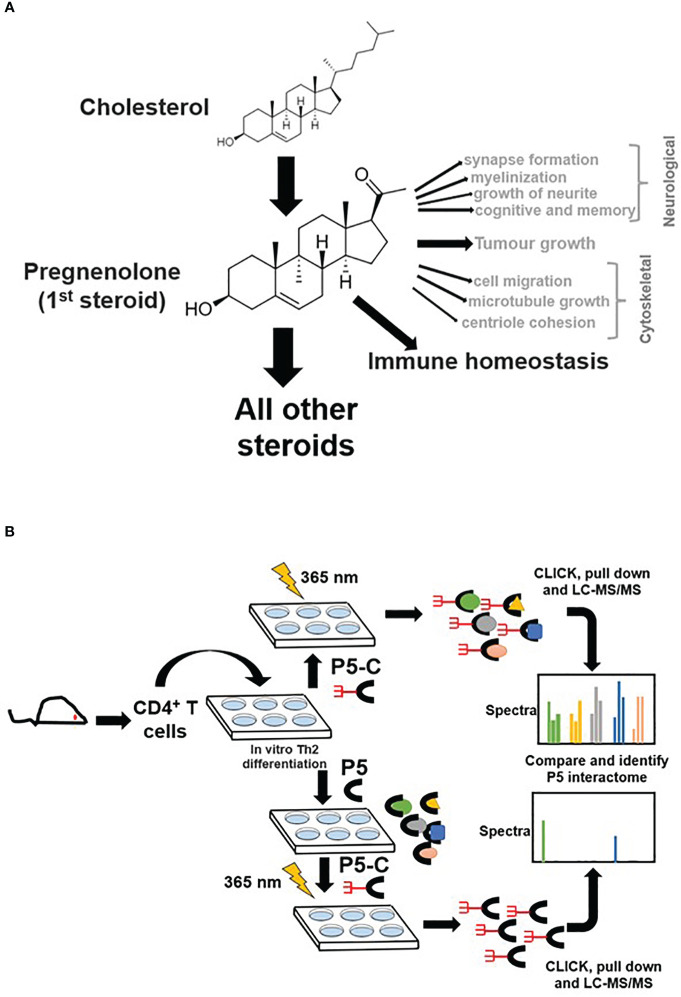
Pregnenolone (P5) and capturing its interactome in CD4+ Th2 cells. **(A)** The pleiotropic function of P5 is depicted in the above schematics. Its role in immune homeostasis has been highlighted that will be primary focus for our study here. **(B)** The schematics describe the capture of P5 interactome in live Th2 cells obtained from mice. The comparison of protein pulldown obtained with or without native P5 provides P5-specific binding proteins from live murine Th2 cells.

**Figure 2:**
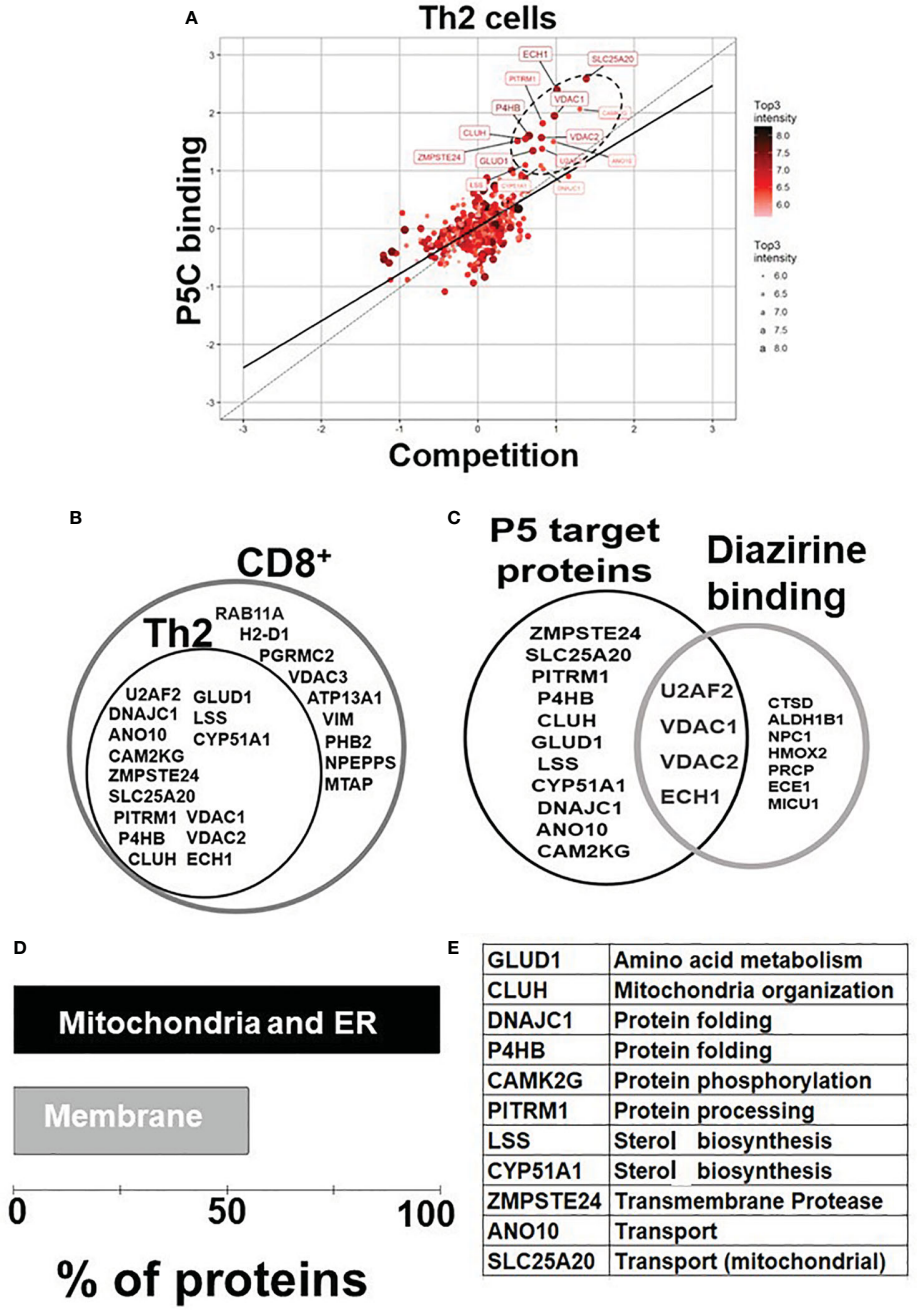
P5 interactome in Th2 cells and their functional analysis. **(A)** Fifteen proteins were significantly enriched as P5 binding in Th2 cells. The proteins extracted with P5-C either alone or after competition with P5. P5-C–interacting proteins were captured from live Th2 cells either in the presence of P5 (competition assay) or in its absence (experiment). The background (empty bead capture) was subtracted from the experiment and competition and then plotted on a log scale to identify P5-binding proteins. The solid straight line represents the regression equation fitting the two variables, and the dotted straight line demonstrates the X = Y linear relation. The solid circles in ellipse with dotted boundary contain the 15 P5-C–binding proteins whose interaction can be competed out in the presence of native P5. **(B)** The diagrammatic representation of the overlap between the P5-binding proteins in two immune cell types: CD8+ T cells and CD4+ Th2 cells. All 15 P5-binding proteins captured in Th2 cells are a subset of proteins from CD8+ T cells. **(C)** The intersection region in the Venn diagram shows four proteins that are known to bind both P5 and diazirine. The 11 specific P5-binding proteins are encircled as “P5 target proteins”. **(D)** Functional categorization of the P5-binding proteins demonstrates a clear enrichment of ER and membranes (~55%) that demonstrate the core functional pathway of P5 in immune cells. **(E)** The table enlisting the GO category “biological process” of all the 11 P5-binding proteins in Th2 cells.

P5 is vital in neurological (7, 38) and cytoskeletal (13, 39) milieu; however, its role in immune homeostasis independently or in the context of the tumor microenvironment is emerging (**Figure 1A**). We used the pregnenolone analog P5-C, whose bioactivity, cell-permeability, and specificity as a mimic of native P5 have been described elsewhere (22). To identify proteins enriched with P5-C in murine immune cells, we used the tandem mass tag (TMT) in conjunction with high-throughput mass spectrometry for relative quantification. **Figure 1B** depicts the succinct plan of identifying P5 interactome from Th2 cells *in vivo*. To select for P5 specificity, P5-C capture was competed with 10X native P5. The proteins that are true P5 binding would be competed out when challenged with native P5. Only significantly enriched proteins that show effective competition when challenged with native P5 were selected. Subsequent analysis revealed 15 P5-interacting proteins enriched in Th2 cells (**Figure 2A**). All these 15 P5-binding proteins from murine Th2 (CD4+) are a subset of the 25 proteins identified to be P5 binding in the CD8^+^ T cells (22) (**Figure 2B**). This reflects the functional conservation of P5 in CD8+ and CD4+ immune cells. To rule out any dual-binding proteins that might have been captured due to the protein’s affinity to the diazirine-alkyne linker, we took advantage of a recent study demonstrating the protein interacting with the diazirine-containing CLICK linker in mammalian cells (40). While comparing, we found four prospective target proteins in our study that also showed dual binding affinity. Although it might be possible that these four proteins can bind P5 and diazirine, we decided to rule out these four common interactors, thereby leading us to specific 11 proteins that are P5 targets in Th2 cells (**Figure 2C**).

### P5-interacting proteins in Th2 cells are localized in mitochondria and endoplasmic reticulum

In agreement with our previous study (22), P5 target proteins in Th2 are predominantly localized in ER, mitochondria, and the membranes. Nine of the 11 (81%) proteins are from the ER and/or mitochondria, and six (55%) of these 11 proteins are on the membranes (**Figure 2D**). Two proteins, PITRM1 (41) and SLC25A20 (42), are localized in the mitochondrion, and glutamate dehydrogenase 1 (GLUD1) is present in both ER and mitochondria, respectively (43). DnaJ heat shock protein family (Hsp40) member C1 (DNAJC1) and zinc metallopeptidase STE24 (ZMPSTE24) are present in ER and nucleus. Clustered mitochondria protein homolog (CLUH) is the only cytoplasmic, and ANO10 is the only plasma membrane protein in the P5-target list. The P5-interacting proteins are involved in cellular functions such as mitochondrial organization, glutamate metabolism, protein folding, transport, and steroid metabolism (**Figure 2E**).

### Molecular docking reveals how P5 interacts with its binding protein

To date, to the best of our knowledge, structural features of P5 binding with its cognate-binding partner proteins are obscure at molecular or submolecular level. We envisage that it would be interesting to understand binding features and the conformational adaptation of the P5–target protein interaction. The substrate P5 is docked with cytochrome P450 family 51 subfamily A member 1 (CYP51A1), lanosterol synthase (LSS), GLUD1, CLUH, prolyl 4-hydroxylase subunit beta (P4HB), and pitrilysin metallopeptidase 1 (PITRM1) protein models through Induced Fit docking (IFD) to evaluate the binding mode. The most important feature of IFD is that the substrate, active site residues of the protein and its vicinity are considered as flexible for better assessment of protein–substrate interactions (31, 32). The IFD results are provided in **Table 1**. The detailed protein–ligand interaction profiler is provided in in **Tables ST1A–F**.

**Table 1:** Docking scores for selected protein models with P5.

Docking Scores		S_Score (kcal/mol)	Rmsd_refine (A°)	E_conf	E_place	E_score1	E_refine	E_score2
P5								
	CLUH	−7.0705	1.2121	20.3313	−41.7799	−7.4497	−38.7172	−7.0705
	CYP51A1	−6.9349	1.4454	16.3240	−60.3607	−9.0636	−31.0231	−6.9349
	GLUD1	−6.9486	1.6890	18.6725	−82.1230	−9.7515	−29.2101	−6.9486
	LSS	−6.0083	1.5237	14.9390	−69.0326	−9.1873	−27.3442	−6.0083
	P4HB	−6.2503	1.1224	44.9485	−59.6411	−9.4770	−19.3375	−6.2503
	PITRM1	−6.0898	1.6296	18.0037	−67.0599	−8.3369	−27.9744	−6.0898

The results are obtained under the following criteria. S score is the final stage of the docking run. RMSD defines the deviation between the pose, in Å, from the original ligand. RMSD refine defines the deviation between the poses before and after the refinement stage. E_conf defines the energy of the conformer at the end of the refinement. Energy is calculated with the solvation option set to Born. Lower final S-scores indicate more favorable poses. The detailed docking result is provided in **Table 1**.

**Figure 3:**
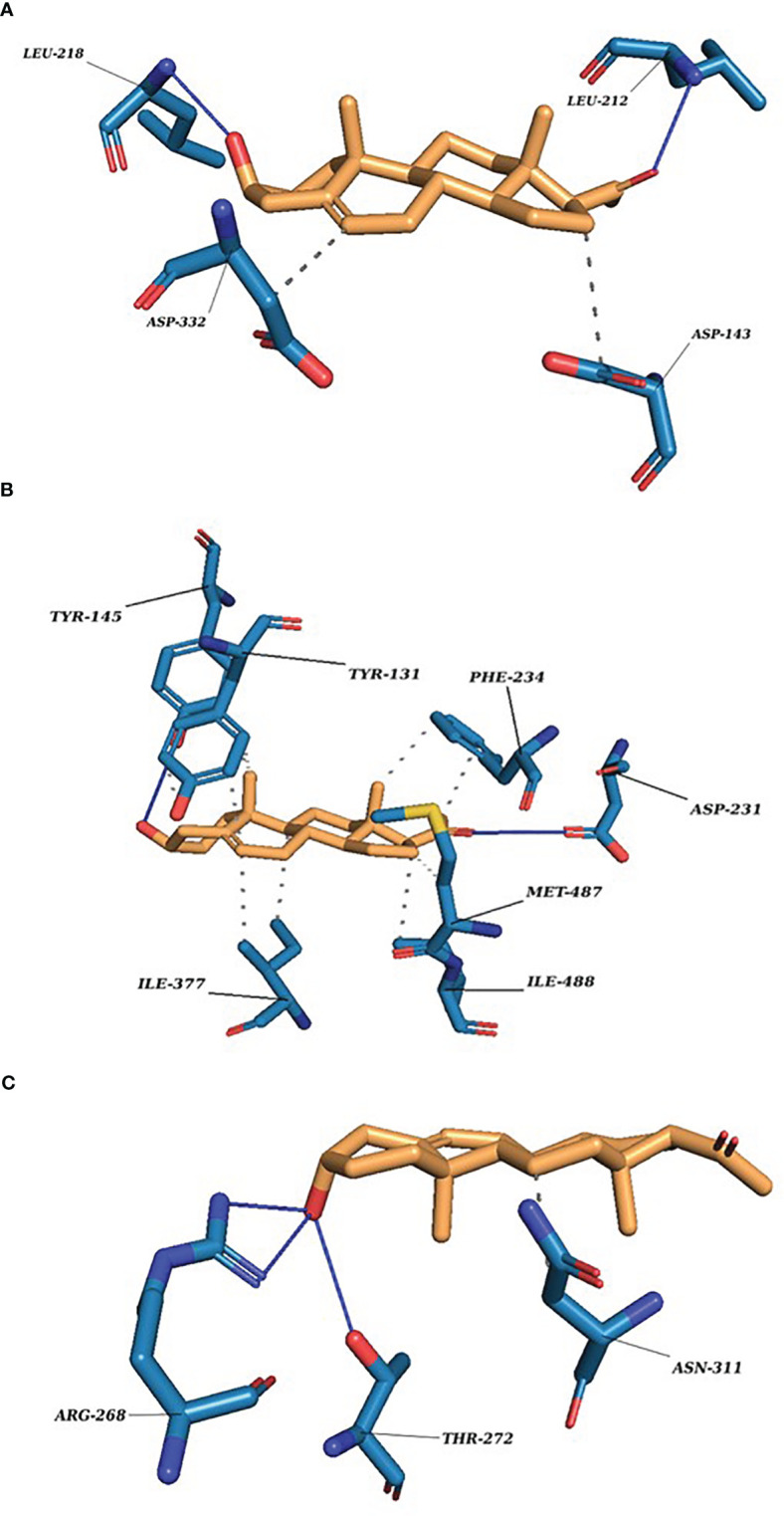
P5 docking complexes. **(A)** CLUH–P5 complex; straight blue lines, hydrogen bonds; dotted blue lines, hydrophobic interactions with the amino acids. **(B)** CYP51A1–P5 complex: straight blue lines, hydrogen bonds; dotted blue lines, hydrophobic interactions with the amino acids. **(C)** GLUD1–P5 complex: straight blue lines, hydrogen bonds; dotted blue lines, hydrophobic interactions with the amino acids.

The docking scores range from −7.0705 to −6.0083 (kcal/mol) for the set of protein–substrate complexes. The root mean square deviation (RMSD) values are within the permissible limits. CLUH, CYP51A1, and GLUD1 showed better binding affinities. The protein–ligand interaction profiler of the selected complex is provided in **Figures 3A-C**. The findings are further validated through molecular simulations and pose rescoring with molecular mechanics/generalized born surface area (MM/GBSA) calculations.

### Evaluation of stability of protein models and their thermodynamic properties

The Desmond module from Schrodinger’s Maestro Suite was implemented for further validation. Several thermodynamic properties for protein and substrate, such as RMSD, root mean square fluctuation (RMSF), radius of gyration (ROG), secondary structure elements (SSEs), protein–substrate timeline, and protein–substrate contacts for CYP51A1, LSS, GLUD1, CLUH, P4HB, and PITRM1 were monitored for 100-ns MD simulation run.

#### Protein RMSD

Protein RMSD values should be within the range of 1–4 Å which indicates that the simulation has equilibrated. Its fluctuation toward the end of the simulation is around thermal average structure. The RMSD values recorded is high for all the proteins because they are homology derived models. The order of protein RMSD values are CYP51A1 (8.8 Å) > CLUH (9.0Å) > P4HB (12.8 Å) > GLUD1 (13.5 Å) > PITRM1 (21.0 Å) > LSS (27.0 Å) (**Figures S1A–F**).

#### Substrate RMSD

The value of substrate RMSD is less than protein RMSD and has stabilized at the end of the simulation for CLUH, CYP51A1, GLUD1, and P4HB protein–substrate complex. This indicates that the substrate is stable with respect to the protein and its binding pocket. If the RMSD values for the substrate observed are significantly larger than the RMSD of the protein, then it is likely that the substrate has diffused away from its initial binding site. The order of substrate RMSD values are CLUH (4.1 Å) > CYP51A1 (4.4Å) > GLUD1 (5.0 Å) > P4HB (14.0 Å) > LSS (19.0 Å) > PITRM (22.0 Å). The detail figure is provided in **Figures S1A–F**.

#### Protein RMSF

The RMSF is useful in characterizing the local changes along the protein chain. On the plots, peaks indicate areas of the protein that fluctuate the most during the simulation. Secondary structural elements like alpha helices and beta strands are usually more rigid than the unstructured part of the protein and thus fluctuate less than the loop regions. Our finding suggests that almost all the proteins have high values of fluctuation in the N and C terminals. The fluctuation was slightly less in CYP51A1 and LSS for C-terminal due to comparative stable SSEs of the proteins (**Figures S2A–F**).

#### Substrate contacts

Protein residues that interact with the substrate are marked with green-colored vertical bars.

#### Substrate RMSF

Substrate RMSF shows the substrate’s fluctuations broken down by atom, corresponding to the two-dimensional structure. The substrate RMSF may give you insights on how substrate fragments interact with the protein residues and their entropic role in the binding event. Our finding shows that LSS and PITRM1 have high entropy values. We assume that this is due to formation of several weak hydrogen bonds and hydrophobic interactions of LSS and PITRM1 proteins with the substrate. The protein–substrate complex is first aligned on the protein backbone, and, then, the substrate RMSF is measured on the substrate heavy atoms (**Figures S3A–F**).

#### Secondary structure elements

The plot provided in **Figures S4A–F** summarizes the SSE composition for each trajectory frame over the course of the simulation, and the plot at the bottom monitors each residue and its SSE assignment over time. Our finding suggests that protein structure had average conformation with 49.03% (CLUH), 55.01% (CYP51A1), 49.52% (GLUD1), 45.13% (LSS), 46.54% (P4HB), and 52.96% (PITRM1) SSE, mainly composed of helices and strands rather than loops and turns that showed conformational changes during MD simulation run (**Figures S4A–F**).

#### Radius of gyration

ROG is analyzed to examine the compactness of the model protein (**Figures S5A–F**).

#### Protein–substrate contacts

Protein–substrate interactions (or “contacts”) are categorized into four types: hydrogen bonds, hydrophobic interactions, ionic bonds, and water bridges (**Figures S6A–F**). The stacked bar charts are normalized over the course of the trajectory: for example, a value of 0.7 suggests that, 70% of the simulation time, specific interaction is maintained. Values over 1.0 are possible as some protein residues may make multiple contacts of the same subtype with the substrate.

Our finding suggests some of the prominent bonds formed by pregnenolone with the following amino acids of the protein: **CLUH:** ARG136, LEU139, LYS140, ASP143, PRO211, LEU212, LEU218, and ASP332. **CYP51A1:** TYR131, PHE234, HIS314, ILE377, MET380, ARG382, HIS447, and CYS449. **LSS:** ASP148, ASN279, PRO283, MET286, TYR287, THR288, PRO289, HIE290, TRP292, HIS295, GLU520, and ARG640. **GLUD1:** LYS147, PRO224, ASP225, THR256, ARG268, THR272, VAL312, ASN431, GLY434, VAL435, and SER438. **PITRM1:** PHE134, ASN136, MET138, TYR380, GLY382, TYR383, SER442, LEU445, THR446, SER449, TYR450, SER453, and SER964. **P4HB:** LYS232, HIS233, GLN235, LEU236, LEU238, ILE240, LYS249, ILE250, LYS285, GLY286, ILE288, LEU289, and PHE290.

Several other bonds and interactions such as ionic bond, hydrophobic interactions, and water channels are also reported. The results were concluded on the basis of protein–substrate contacts bar diagram and residue decomposition values. We have considered the bonds keeping in mind all the possible substrate conformations and poses generated during the simulation run. The detailed representation is provided in **Figures S6A–F**, **Table ST2**, and Residue Decomposition_P5.xlxs.

#### Protein–substrate timeline

A timeline representation of the interactions and contacts (H-bonds, hydrophobic, ionic, water bridges) is summarized (**Figures S7A–F**). The top panel shows the total number of specific contacts that the protein makes with the pregnenolone over the course of the trajectory. The bottom panel shows which residues interact with the substrate in each trajectory frame. Some residues make more than one specific contact with the pregnenolone, which is represented by a darker shade of orange, according to the scale to the right of the plot (**Figures S7A–F**).

#### Pose rescoring with MMGBSA

The conformations of the docking results and substrate efficiency were determined by calculating binding free energy through Prime MM/GBSA (Prime, Schrodinger, LLC, New York, NY, 2022). MM/GBSA scores usually provide a significant correlation with experimentally determined data. The calculated binding free energy for the docked complexes using the all-atom optimized potentials for liquid simulations (OPLS-AA) force field and the GBSA continuum solvent are presented in **Table 2**. The binding free energy for the protein–substrate ranges from −27.29kcal/mol to −58.35kcal/mol. The substrate molecule pose is considered as the best that utilizes less energy to comfort inside the active pocket of the protein. In our study CLUH, CYP51A1, and P4HB showed better rescoring binding energy values. The results are an improvement over IFD results where the finding shows that CLUH, CYP51A1, and GLUD1 showed better binding affinity values. MM/GBSA calculation classified the binding affinity of the substrate in the exact place as docking energy while there were little changes in the order for intermediate docking energy holding compounds (**Table 2**).

**Table 2:** MM/GBSA values for protein models with P5.

S. No.	Proteins	ΔG Bind	ΔG Bind Coulomb	ΔG Bind vdw	ΔG Bind Covalent	ΔG Bind Solv GB	ΔG Bind Lipo	ΔG Bind H Bond
1	CLUH	−58.35616818	−12.22089589	−47.73719881	0.452964344	18.88040592	−16.85542031	−0.876023442
2	CYP51A1	−57.6212031	−8.961990524	−48.5034085	1.316226552	25.35348989	−26.06089958	−0.764620935
3	GLUD1	−29.17202635	−4.748506696	−36.82294515	1.397948969	22.92806308	−11.28422616	−0.642360396
4	LSS	−28.56342559	−4.33566621	−24.56548335	0.760308397	11.32898609	−11.53867904	−0.212891473
5	P4HB	−57.04931056	−17.12902166	−46.02070591	1.835749835	23.05314964	−17.71682611	−1.071656346
6	PITRM1	−27.29123782	−4.965643417	−2540774676	0.66446038	15.3832475	−12.73605131	−0.229504201

Finally, to measure the contribution of the individual residues, the MM/GBSA decomposition analysis was performed. MD simulation analyses of the substrate-residue interactions in the above section are based on the trajectory averaged and minimized, i.e., on static protein–substrate conformations, the MD-based decomposition considers the contribution of protein residues in all possible binding modes (Residue Decomposition_P5.xlxs).

CaverDock is a tool that uses Caver, to identify tunnels in protein structures. It is an optimized version of the well-established algorithm from AutoDock Vina to calculate possible ligand trajectories along those tunnels and the corresponding binding energies.

**Figure 4:**
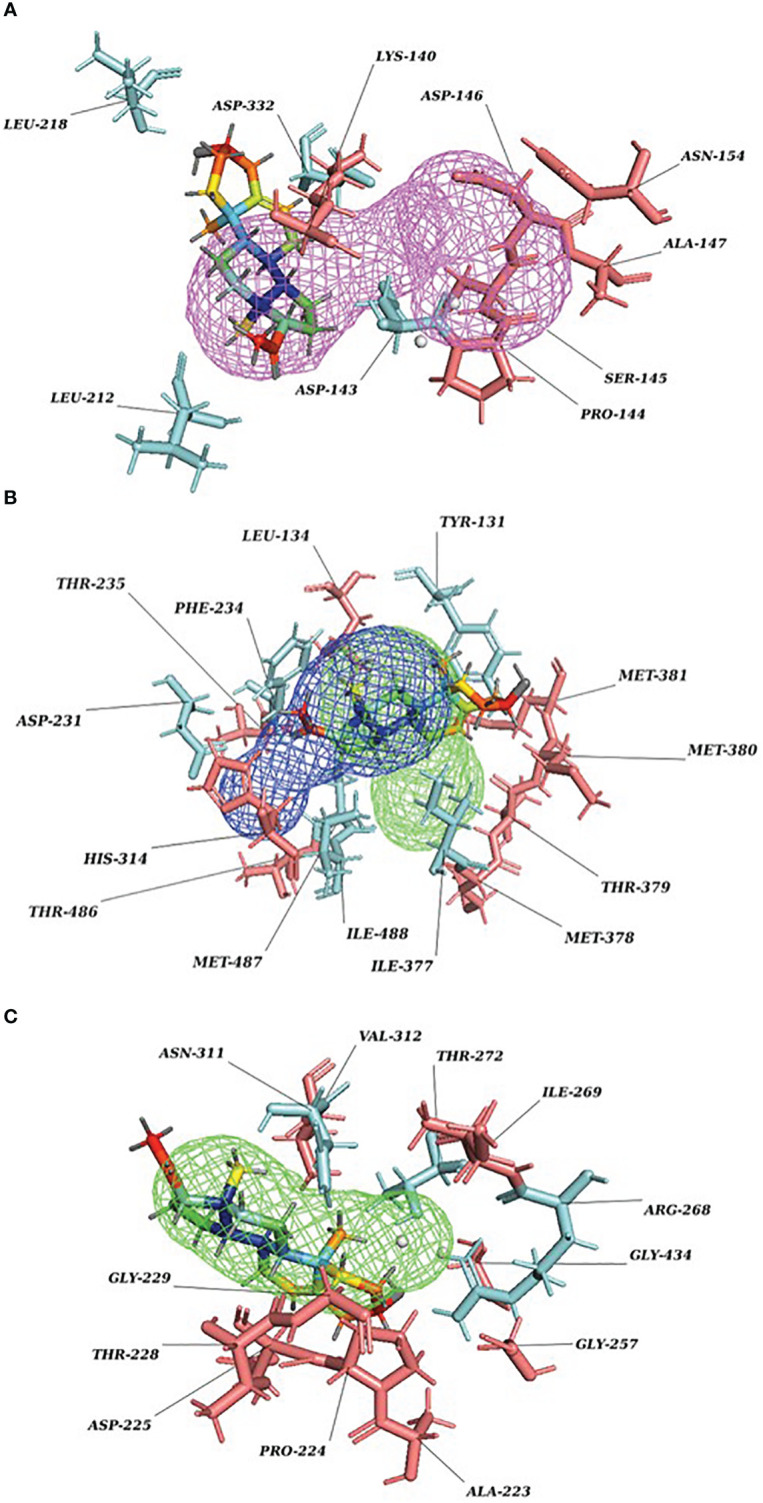
P5 transport through protein tunnel and channel. **(A)** Substrate P5 transport for CLUH protein through Tunnel 6. Cyan color denotes hydrogen and hydrophobic interactions; salmon red color denotes other amino acids surrounding the tunnel. **(B)** Substrate P5 transport for CYP51A1 protein through Tunnel 2. Cyan color denotes hydrogen and hydrophobic interactions; salmon red color denotes other amino acids surrounding the tunnel. **(C)** Substrate P5 transport for GLUD1 protein through Tunnel 2. Cyan color denotes hydrogen and hydrophobic interactions; salmon red color denotes other amino acids surrounding the tunnel.

**Figure 5:**
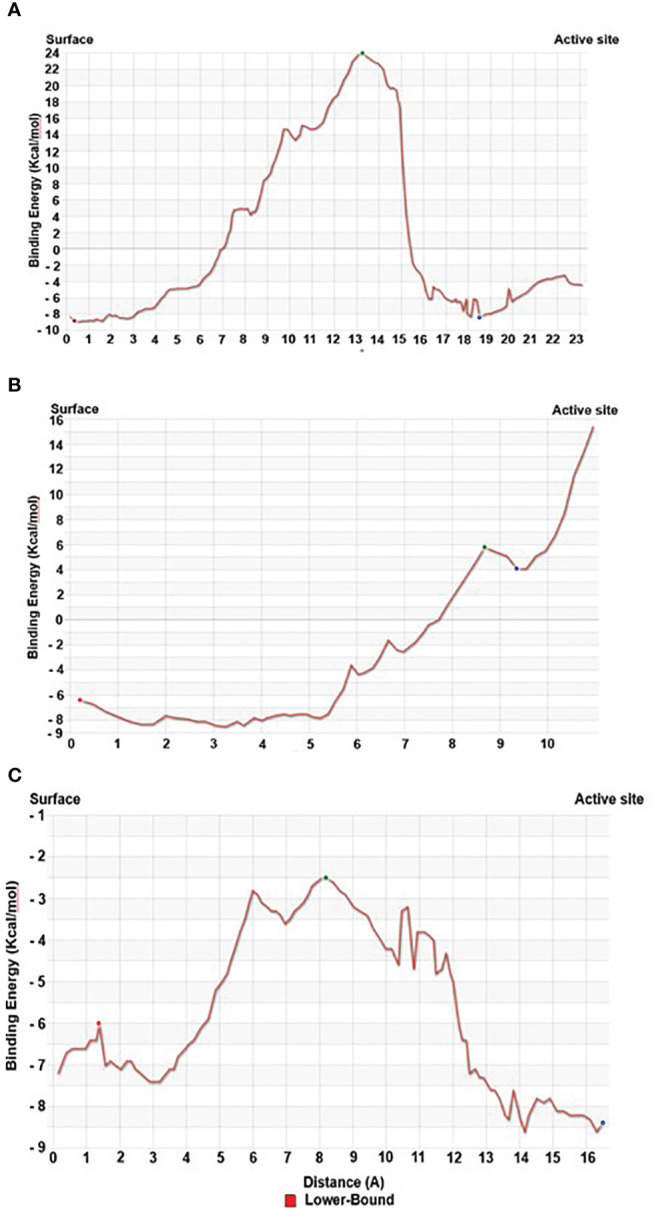
Energy profile of tunnel for proteins. **(A)** Energy profile of Tunnel 6 for CLUH protein [red dot represents E_Surface_ (kcal/mol), green dot represents E_Max_ (kcal/mol), and blue dot represents E_Bound_ (kcal/mol)]. **(B)** Energy profile of Tunnel 2 for GLUD1 protein [red dot represents E_Surface_ (kcal/mol), green dot represents E_Max_ (kcal/mol), and blue dot represents E_Bound_ (kcal/mol)]. **(C)** Energy profile of Tunnel 2 for CYP51A1 protein [red dot represents E_Surface_ (kcal/mol), green dot represents E_Max_ (kcal/mol), and blue dot represents E_Bound_ (kcal/mol)].

Our Caver study confirms that substrate pregnenolone interacts well with important bottleneck amino acid residues in the tunnel with GLUD1, CYP51A1, and CLUH proteins (**Figures 4A–C**, **Table ST3**). Bottleneck residues are marked (salmon red) in bold for the respective proteins that are involved in the interaction with pregnenolone. Tunnel 2 (GLUD1-ARG268, THR272, and ASN311), Tunnel 2 (CYP51A1-TYR131, ASP231, PHE234, ILE377, and ILE488), and Tunnel 6 (CLUH-ASP143) show hydrogen bonds and hydrophobic interactions with the pregnenolone (**Tables ST3, ST4**). The energy profile of the tunnels for respective enzymes has been performed by selecting the correct tunnel that allows substrate to transport. We can observe that, after passing through the bottleneck residues, pregnenolone shows selective binding at the active site for CYP51A1 and CLUH enzyme with a favorable binding energy of −8.4 kcal/mol for both the proteins, respectively. The energy barrier around the gateway residues was lower for both CYP51A1 (−8.8 (kcal/mol, Tunnel 1) and (−2.4(kcal/mol, Tunnel 2) and CLUH (0.4 kcal/mol, Tunnel 6), respectively (**Table 3**). Pregnenolone interaction with GLUD1 shows slightly higher binding energies (4.1 kcal/mol). The difference of the binding energy of pregnenolone in the active site and at the surface for the gateway residues for pregnenolone–GLUD1 complex was recorded high (10.5 kcal/mol). The detailed CaverDock result in form of energetic profile is provided in **Figures 5A-C**. The substrate does not pass through tunnels for proteins LSS, P4HB, and PITRM1 (**Figures S8A–C**), and the binding of pregnenolone with the amino acids takes place outside the tunnels. We further used alanine scanning to show the importance of key amino acids found in our interaction studies in overall functioning of the proteins. For this, we selected the top three candidates from our previous MM/GBSA (**Table 2**) analysis (CLUH, P4HB, and CYP51A1). Our findings clearly show that **dStability** values for the wild type is better than that for the mutant type described for the selected amino acids obtained from the molecular modeling studies for CLUH, P4HB, and CYP51A1 (**Tables 4A, B**, **5A, B**, **Tables ST5–ST9**). The involvement of important amino acid in the binding pocket played a key role in binding with the substrate.

**Table 3:** Energy profile of substrate transport within the tunnel for GLUD1, CYP51A1, and CLUH protein–substrate complex.

Substrate	Tunnel	Direction	E_Bound_ (kcal/mol)	E_Max_ (kcal/mol)	E_Surface_ (kcal/mol)	E_a_ (kcal/mol)	ΔE_BS_ (kcal/mol)
GLUD1_P5	2	IN	4.1	5.8	−6.4	12.2	10.5
CYP51A1_P5	1	IN	−7.3	3.5	1.5	2.0	−8.8
CYP51A1_P5	2	IN	−8.4	-2.5	−6.0	3.5	−2.4
CLUH_P5	6	IN	−8.4	24.0	−8.8	32.8	0.4

**Table 4(A):** MM/GBSA values for Human sterol 14a-demethylase (CYP51) in complex with the substrate ketoconazole with P5 (3LD6).

S. No.	Proteins	ΔG Bind	ΔG Bind Coulomb	ΔG Bind vdw	ΔG Bind Covalent	ΔG Bind Solv GB	ΔG Bind Lipo	ΔG Bind H Bond
MM/GBSA								
**1.**	**3LD6(CYP51A1-Abiraterone)**	**−51.60993134**	**−5.647333075**	**−50.90847789**	**0.790975017**	**41.70110837**	**−35.75468545**	**1.66E-01**
**2.**	**3LD6(CYP51A1-Pregnenolone)**	**−39.38632504**	**−11.0731566**	**−45.67248827**	**0.9110947703**	**44.54417216**	**−27.53911235**	**−0.556687686**

**Table 4(B):** Selected amino acids from Residue decomposition analysis for human sterol 14a-demethylase CYP51 in complex with the substrate ketoconazole with P5 3LD6.

S. No	Proteins	Amino Acid (Hydrophobic Bonds)	Hydrogen Bonds	Ionic Bonds	Pi-Pi
**1.**	**3LD6(CYP51A1-Abiraterone)**	**Phe77, Tyr131, Leu134, Phe234, His236, Trp239, Ile377, Ile379, Met381**	**Thr135, Trp239**	**THR135, HEM601**	**PHE77, PHE105, HIS236, TRP239**
**2.**	**3LD6(CYP51A1-Pregnenolone)**	**Tyr131, Leu134, Val138, Phe139, Lys156, Phe234, Ala311, Ile377, MET380**	**Ile379, Met487**	**LYS156, ILE379, MET487, HEM601**	**NA**
**3.**	**AlphaFold_** **Pregnenolone**	**TYR131, PHE234,** **HIS314, ILE377, MET380, CYS449**	**HIS314, ARG382, HIS447**	**HIS447, ARG382, CYS449**	**NA**

NA, Not applicable.

## Discussion

Although P5’s importance as a bioactive molecule with pleiotropic functions is beyond doubt, its biochemical mode of action is not fully understood. P5 demonstrates crucial functions in regulation of inflammation and immunity (12, 15, 16, 44). CD4+ Th2 and CD8+ subtypes of immune cells produce P5 via local steroidogenesis (15, 16). We have deciphered P5 interactome in the CD8+ cells (22) and P5 interactome in Th2, and a CD4+ subtype would reveal conserved and specific pathways of P5 biochemistry in these immune cells. With this aim, we sought out to find P5-interacting proteins specific to Th2 cells. We envision that our finding could provide a comprehensive biochemical underpinning of P5 that are concurrent and distinct to immune cell types.

The bioactive and cell-permeable CLICK-based P5 analog (22) was used to mimic native P5 in live Th2 cells. In conjunction with high-throughput mass spectrometry, we revealed a comprehensive proteome-wide interactome map of P5 in Th2 immune cells. We identified 11 P5-binding proteins in Th2 cells as specific P5-interacting proteins. Interestingly, all these 11 proteins are a subset of the 25 P5-binding proteins that were previously identified in CD8+ cells by us (22). This suggests conservation of key P5 function across CD4+ and CD8+ immune cell types. We envisage that these proteins may represent the core conserved functional pathways regulated by P5 in immune cells. The identified proteins were from mitochondria and endoplasmic reticulum (>80%) and membranes (~55%) (**Figure 2D**), which demonstrated a non-genomic mode of P5 activity corroborated by many previous studies (5, 13, 14, 22, 39).

Mitochondria (45) and ER (46) are known to play crucial roles in immune cells. P5 target proteins localized in these organelles suggests P5’s regulatory role in modulating Th2 cells. In our previous study, we found mitochondria acyl-carnitine transport pathway proteins as P5 targets in CD8+ cells (22). In CD4+ cells, we found the mitochondrial SLC25A20, the transporter of the acyl-carnitine, to be a target of P5. P5 is synthesized in mitochondria (2), and we found CLUH to be a target of P5. CLUH is an RNA-binding protein that controls mitochondrial biogenesis and distribution by modulating the expression of nuclear-encoded mitochondrial RNAs (47). It is, therefore, possible that P5 can regulate mitochondrial homeostasis via CLUH. Understanding the structural details of P5-CLUH molecular interaction can provide useful insight of this function. Mitochondrial GLUD1, another P5 target, is known to play key role in replenishing tricarboxylic acid (TCA) cycle intermediate α-ketoglutarate from L-glutamate (48). α-Ketoglutarate performs crucial role in T-cell differentiation (49). Furthermore, GLUD1 enzyme plays an important role in insulin balance (50) and is one of the top candidates that is involved in long term memory and spatial learning (51). P5’s role in the nervous system is well-known (5), and GLUD1 may be the entry point to comprehend the biochemical pathway of P5 activity in brain cells. Steroid and their derivatives have a variety of roles in regulation of immunity mediated by the different immune cell types (52). Their roles are still emerging; in this context, we found two key enzymes, CYP51A1 and LSS, in the sterol biosynthetic pathway to be P5 targets. This suggests a regulatory role of P5 in the biosynthesis of sterol, including cholesterol, the immediate parent molecule from which P5 is synthesized. P5 regulating its own homeostasis within steroidogenic cells via feedback inhibition seems plausible. Other targets of P5 are related to cellular and sub-cellular transport and protein processing and folding. All these functions are crucial in regulation of the immune system.

We further provided insight into the molecular interaction of P5 and its key targets at the atomic level. To do this, we employed state-of-the art docking, molecular dynamics, and tunnel and channel detection tools. The docking results clearly indicate binding up of proteins with the substrate pregnenolone. The values are comparable, and the RMSD values are within the range of 2Å. CLUH, CYP51A1, and GLUD1 protein shows better binding affinity with the substrate pregnenolone. The docking results are well complemented by the simulation findings. RMSD values of the modeled protein backbone are higher, but the substrate fits the binding site. The NPT (number of particles, pressure, and temperature) ensemble system has equilibrated until the end of the simulation run. The exception was recorded for PITRM1 protein (**Figures S1A–F**). Protein RMSF was recorded for all the proteins (**Figures S2A–F**). The property is useful in characterizing the local changes along the protein chain. All the proteins under investigation constitute mainly of alpha helices and beta sheets (**Figures S4A–F**). CYP51A1 and LSS recorded the least fluctuation of amino acids among all the proteins under investigation (**Figures S2A–F**). The substrate RMSF values for proteins LSS and PITRM1 have high entropy values. This is due to the formation of several weak hydrogen bonds and hydrophobic interactions (**Figures S3A–F**). All the proteins did not attain the compactness throughout the simulation run (**Figures S5A–F**). Substrate–protein contacts clearly show that P4HB forms the strongest H-bonds followed by CLUH and CYP51A1 (Residue Decomposition_P5.xlsx). MM/GBSA studies were performed to study the correlation of *in silico* findings with experimentally determined data. The simulation analysis performed is based on the trajectory averaged and minimized, i.e., on static protein–substrate conformations. MM/GBSA considers the trajectory output generated in the simulation run to further validate the docking findings. The main purpose of the study is to look deep into the energy values that utilize less energy for the substrate to comfort inside the active pocket of the proteins. The study reveals that CLUH, CYP51A1, and GLUD1 showed better binding affinity values while taking into special consideration of intermediate docking energies (**Table 2**). Finally, we measured the contribution of the individual residues in the interaction with substrate through residue decomposition analysis. This analysis considers the contribution of protein residues in all possible binding modes. The result provides deep insight into the possible bond formation of the substrate complexes in the form of binding energies, Coulombic, hydrogen bonds, van der Waals, and lipophilic and hydrophobic interactions (Residue Decomposition_P5.xlxs).

Protein forms functionally important local substructures, such as active sites, allosteric sites, tunnels, and channels. The tunnels are mainly present in globular proteins with catalytic function (enzymes) and serve as the access pathways for substrates, products, co-factors, water molecules, and/or inhibitors from a bulk solvent to buried active sites. Our study reveals that pregnenolone interacts with important bottleneck amino acid residues in the tunnel with GLUD1, CYP51A1, and CLUH proteins (**Figures 4A-C**). The energy profile analysis for the tunnels for respective proteins has been performed to select the correct tunnel that allows substrate to transport (**Table 3**). The various parameters are summarized as follows: Tunnel, selected protein tunnel; Substrate, name of the used substrate; Direction, direction of the CaverDock calculation; E_Bound_, the binding energy of the substrate located in the binding site; E_Max_, the highest binding energy in the trajectory; E_Surface_, the binding energy of the substrate located at the protein surface; E_a_, activation energy of association, E_Max_–E_Bound_ for products and E_Max_–E_Surface_ for reactants (describes the difficulty of getting through the tunnel; kinetics); ΔE_BS_, difference of the binding energies of the substrate in the active site and at the surface (corresponds to enthalpy; thermodynamics).The CaverDock result shows the energetic profile of the substrate binding. The graph generated for the respective tunnel allows us to select the values that correspond to the bound state (E_Bound_), maximum energy (E_Max_), and unbound state (E_Surface_). After selecting these three points, CaverDock automatically calculates the estimate of the binding energy barrier (E_A_) and the difference in energy of the bound and unbound states (E_BS_). To study the ease of access to the druggable site for the substrate, E_Max_ (the highest binding energy in the trajectory) and E_a_ (activation energy of association: E_Max_–E_Surface_ of the reactants) are two parameters of importance. To select well binding substrates, we should aim for the lowest values in both E_Max_ and E_a_ in terms of the energy values (**Table 3**). In our study, for GLUD1 and CLUH, only one tunnel is being reported for substrate transport. In case of CYP51A1, two tunnels are reported. It can be seen from **Table 3** that the values of E_a_ are comparable for both the tunnels, but Tunnel 2 is comparatively stable for CYP51A1 to transport substrate than Tunnel 1 due to lower value of E_Max_. The detailed representation of the energy profile for the selected protein-complex is provided in **Tables ST3, ST4** and **Figures 5A-C**. The substrate does not pass through tunnels for LSS, P4HB, and PITRM1 protein complexes (**Figures S8A–C**). The binding of substrates for the above-mentioned proteins take place outside the active sites. We took the top three candidate proteins and performed alanine scanning to show the importance of the key P5-interacting amino acid in the overall functioning of the proteins (**Tables 4**, **5**, **Tables ST5–ST9**).

**Table 5(A):** MM/GBSA values for Human sterol 14a-demethylase (CYP51) in complex with the substrate lanosterol with P5 (6UEZ).

S. No.	Proteins	ΔG bind	ΔG Bind Coulomb	ΔG Bind vdw	ΔG Bind Covalent	ΔG Bind Solv GB	ΔG Bind Lipo	ΔG Bind H Bond
MM/GBSA								
**1.**	**6UEZ(CYP51A1-Lanosterol)**	**-56.69293565**	**−8.83758**	**−68.87617465**	**2.263307908**	**66.85053637**	**−47.54190985**	**−0.551116006**
**2.**	**6UEZ(CYP51A1-Pregnenolone)**	**-57.72292237**	**−7.897273578**	**−67.55987562**	**1.58601087**	**62.25752417**	**−45.56100073**	**−0.548307486**

**Table 5(B):** Selected amino acids from residue decomposition analysis for human sterol 14a-demethylase CYP51 in complex with the substrate lanosterol with P5 3LD6.

S. No	Proteins	Amino Acid (Hydrophobic Bonds)	Hydrogen Bonds	Ionic Bonds
**1.**	**6UEZ(CYP51A1-Lanosterol)**	**TYR131, LEU134, ALA144, TYR145, PHE152, LEU159, PHE234, MET304, LEU310, ALA311, ILE377, MET487, ILE488**	**ILE379**	**PHE234, ILE377, PHE139, HEM601**
**2.**	**6UEZ(CYP51A1-Pregnenolone)-(Chain-B)**	**TYR131, LEU134, PHE139, ALA144, PHE152, PHE234, LEU310, ALA311, HIS314, ILE377, ILE450, ILE488**	**ILE379**	**TYR131, PHE234, ILE377, HEM601**
**3.**	**AlphaFold_** **Pregnenolone**	**TYR131, PHE234,** **HIS314, ILE377, MET380, CYS449**	**HIS314, ARG382, HIS447**	**HIS447, ARG382, CYS449**

P5’s role as a lymphosteroid has not been investigated in detail. Our study not only revealed the specific P5-interacting proteins in live murine Th2 cells but also identified near-native key P5–protein molecular interactions. We found that mitochondrial and ER localized proteins play key roles in mediating P5 activity in CD4+ and CD8+ immune cells. Some of the P5-binding proteins identified from our study, such as GLUD1, PITRM1, P4HB, and CYP51A1 are already well-known therapeutic targets for drug development. We point out that our study has unlocked a new domain to explore the mechanism of P5’s mode of action in immune cells that will provide insights into designing innovative drug targets from the molecular dynamics output of P5–protein interactions.

## Materials and methods

### Materials

The P5 analog P5-C has been described previously. The probe is bioactive and cell-permeable and has the capacity to specifically capture P5-binding proteins.

### Th2 cell proliferation

The detailed method is described previously (22).

### P5-binding proteomics on murine Th2 cells

Ten million murine CD4+ Th2 immune cells were used in three different experiments with two replicates for each. In the first experiment, Th2 cells in phenol free Roswell Park Memorial Institute (RPMI) medium were incubated with only the vehicle (without probe), second experiment had 10 µM P5-C, and the third was incubated with 100 µM (10X) of P5 before 10 µM of P5-C incubation. The following protocol was the same for all the experiment and its replicates. The cells were centrifuged, the medium was removed, and cells were washed once with cold PBS and then irradiated at 365 nm for 15 min in 200 μL of cold PBS at 4°C. Subsequently, the Phosphate Buffered Saline (PBS) was removed after centrifugation, and the cells were washed once with ice cold PBS. Lysis of Th2 cells were done in PBS containing 0.2% Sodium Dodecyl Sulphate (SDS). Lysates were centrifuged, and the supernatant containing the clear lysates was used for copper click and affinity purification. Following three times PBS/0.1% SDS, the beads were washed three times with 1 mL of PBS. The washed neutravidin beads were sent to European Molecular Biology Laboratory (EMBL) proteomics core facility for TMT labeling, peptide fractionation, and mass spectrometry.

### Mass spectrometry

#### Sample preparation and TMT labeling

The samples were prepared the same way as described before. Briefly, 20 µL of 4× Laemmli buffer was added to ~60 µL of beads after removal of supernatant, followed by vortexing and then kept for shaking in a thermal mixer for 15 min at 95°C, and subsequently followed by another round of vortexing and shaking for additional 15 min at 95°C. After cooling the samples to room temperature, they were filtered using a 90-µm Mobi column filter. To reduce disulfide bridges, 25 µL of the remaining was first diluted with 50 µL of 50 mM 4-(2-hydroxyethyl)-1-piperazineethanesulfonic acid (HEPES) solution and then treated with 2 µL of 200 mM Dithiothreitol (DTT) in 50 mM HEPES at pH 8.5 for 30 min at 56°C. Carbamidomethylation of the accessible cysteine residues was done by adding 4 µL of 400 mM 2-chloroacetamide in 50 mM HEPES at pH 8.5 and incubation for 30 min in the dark.

Protein clean-up and digestion were done by two single-pot solid-phase-enhanced sample preparations (SP3) (53). Sera-Mag Speed Beads (ThermoFisher) was prewashed with water, and 2 µL of a 1:1 mixture of the hydrophilic and hydrophobic beads was added to each sample at a concentration of 10 µg/µL. Acetonitrile (83 µL) was added to each sample, and the suspensions were incubated for 8 min; subsequently, the vials were kept on the magnetic stand for 2 more min. After discarding the supernatants, the beads were washed twice with 200 µL of 70% ethanol and once with 180 µL of acetonitrile. After discarding the acetonitrile, the beads were air-dried. Trypsin (150 µg) in 10 µL of 50 mM HEPES buffer was added to the beads, and the bead-bound proteins were digested for overnight. After incubation, on the next day, the bead suspensions were sonicated for 5 min and vortexed after which the vials were kept on the magnet. The supernatants containing the peptides were transferred to new vials. The magnetic beads were rinsed with 10 µL of 50 mM HEPES buffer, and the resulting supernatants were combined with the first 10 µL. Following that, individual samples were labeled by adding 4 µL TMT-6plex reagent (ThermoFisher) in acetonitrile, which were then incubated for 1 h at room temperature. After 1 h, the reactions were quenched with a solution of 5% hydroxylamine, and, then, the mixture was acidified with 50 µL of 0.05% formic acid. The samples of obtained from each replicate were cleaned using an Oasis hydrophilic lipophilic balance (OASIS HLB) µElution Plate (Waters). The wells were first washed twice with 0.05% formic acid in 80% acetonitrile and twice with 0.05% formic acid in water. The samples were loaded on the wells and washed with 0.05% formic acid in water. After elution with 0.05% formic acid in 80% acetonitrile, the samples were dried and reconstituted in 4% acetonitrile and 1% formic acid in water.

#### Peptide fractionation

The pH of the samples was adjusted to 10 with ammonium hydroxide. The TMT-labeled peptides were then fractionated on an Agilent 1200 Infinity HPLC system equipped with a degasser, quaternary pump, autosampler, a variable wavelength UV detector (that was set to 254 nm), and fraction collector. Separation was performed on a Phenomenex Gemini C18 (100 mm × 1.0 mm; 3 μm; 110 Å) column using 20 mM ammonium formate at pH 10 in water as mobile phase A and 100% acetonitrile as mobile phase B. The column was used in combination with a Phenomenex Gemini C18, 4 mm × 2.0 mm Security Guard cartridge. The flow rate was 0.1 mL/min. After 2 min of isocratic separation at 100% A, a linear gradient to 35% B at minute 59 was used, followed by washing at 85% B and reconstitution at 100% A. In total, 32 2-min fractions were collected and pooled. These were dried and reconstituted in 4% acetonitrile and 1% formic acid.

#### Mass spectrometry data acquisition

The fractionated samples were analyzed on an UltiMate 3000 nano LC system (Dionex) coupled to a QExactive plus (Thermo) mass spectrometer via a Nanospray Flex source (Thermo) using a Pico-Tip Emitter (New Objective; 360-µm Outside Diameter (OD) × 20-µm Inside Diameter (ID); 10-µm tip). The peptides were first trapped on a C18 PepMap 100 µ-Precolumn (300 µm × 5 mm, 5 µm, 100 Å) prior to separation on a Waters nanoEase C18 75 µm × 250 mm, 1.8 µm, 100 Å column. The applied flow rates were 30 µL/min for trapping and 300 nl/min for separation. The mobile phase A was 0.1% formic acid in water, and the mobile phase B was 0.1% formic acid in acetonitrile. After an initial isocratic step at 2% B for 2.9 min, the multi-step gradient started with a gradient to 4% B at minute 4 followed by a linear increase to 8% B at minute 6. Subsequently, a shallow gradient to 28% B at minute 43 was followed by a steep gradient to 40% B at minute 52, a washing step at 80% B, and reconstitution at 2% B.

All spectra were acquired in positive ion mode. Full-scan spectra were recorded in profile mode in a mass range of 375–1,200 mass-to-charge ratio (*m/z*), at a resolution of 70,000, with a maximum ion fill time of 10 ms and an Automatic Gain Control (AGC) target value of 3 × 10 (6) ions. A top 20 method was applied with the normalized collision energy set to 32, an isolation window of 0.7, the resolution at 17,500, a maximum ion fill time of 50 ms, and an AGC target value of 2 × 10 (5) ions. The fragmentation spectra were recorded in profile mode with a fixed first mass of 100 *m/z*. Unassigned charge states and charge states of 1, 5–8, and >8 were excluded, and the dynamic exclusion was set to 30 s.

### Analysis of the MS results

#### MS data analysis for murine Th2 cells

IsobarQuant (54) and Mascot (v2.2.07) were used to process the acquired data, which was searched against the UniProt reference database of *Mus musculus* after the addition of common contaminants and reversed sequences. The following modifications were included into the search parameters: carbamidomethyl (C) and TMT10 (K) (fixed modification), acetyl (N-term), oxidation (M), and TMT10 (N-term) (variable modifications). A mass error tolerance of 10 parts per million (ppm) was applied to full-scan spectra and 0.02 Da to fragmentation spectra. Trypsin was selected as protease with an allowance of maximum two missed cleavages and a minimum peptide length of seven amino acids, and at least two unique peptides were required for protein identification. The false discovery rate (FDR) on peptide and protein level was set to 0.01.

The protein.txt output files from IsobarQuant were further processed using the R language. As quality filters, only proteins that were quantified with at least two unique peptides and have been identified in both biological replicates (analyzed in separate Mass Spectrometry (MS) analysis) were used for further downstream analysis. The “signal_sum” columns were used, and potential batch effects were removed using the respective function from the limma package (55). Subsequently, the data were normalized using a variance stabilization normalization (56). The limma package was employed again to test for differential abundance between the various experimental conditions. T-values of the limma output were pasted into fdrtool (57) to estimate FDRs (q-values were used as FDR).

### Homology modeling

The current study has been performed on six different protein targets. These are CYP51A1 (UniProt entry Q8K0C4), LSS (Q8BLN5), GLUD1 (P26443), CLUH (Q5SW19), P4HB (P09103), and PITRM1 (Q8K411). The substrate selected is Pregnenolone (Pubchem compound Compound Identifier (CID): 8955). We have obtained our AlphaFold modeled structures for all the proteins from the UniProt database. AlphaFold (58, 59) is a novel machine learning approach that incorporates physical and biological knowledge about protein structure, leveraging multi-sequence alignments, into the design of the deep learning algorithm. The state-of-art artificial intelligence (AI) system developed by DeepMind can computationally predict protein structures with unprecedented accuracy and speed. AlphaFold generated models have accuracy around 93% and can be used for purposeful drug designing related projects. AlphaFold identifiers selected for our studies are CYP51A1 (AF-Q8K0C4-F1), LSS (AF-Q8BLN5-F1), GLUD1 (AF-P26443-F1), CLUH (AF-Q5SW19-F1), P4HB (AF-P09103-F1), and PITRM1 (AF-Q8K411-F1).

### Molecular docking

AlphaFold protein models for CYP51A1, LSS, GLUD1, CLUH, P4HB, and PITRM1 were prepared in MOE2022.02 software [Molecular Operating Environment (MOE), 2022.02; Chemical Computing Group ULC, 1010, Sherbooke St. West, Suite #910, Montreal, QC, Canada, H3A 2R7, 2022]. The water molecules and heteroatoms were removed, and polar hydrogens were added. A temperature of 300 K, salt concentration of 0.1, and pH 7 were quantified in an implicit solvated environment to undergo the protonation process. The structure was energy minimized in the Amber10: EHT force field to an RMS gradient of 0.01 kcal/mol/A (2). The energy minimized conformation of protein models was then subjected to 10-ns molecular dynamic simulations at a constant temperature of 300 K, heat time of 10 ps, and temperature relaxation of 0.2 ps to derive a stable conformation.

The “Site Finder” feature on MOE 2022.02 was used to predict the active sites of the protein models. We have created a dummy model out of the generated active sites to derive the binding site of the modeled proteins. We have considered the largest active site based on participation of maximum amino acids for our docking studies. The docking process was conducted three times. The first and second docking experiment was performed by using “Rigid Receptor” protocol. In this simulation, we considered “Triangle Matcher and London dG” (60) as placement method and scores, respectively. Similarly, “Rigid receptor/Induced Fit and GBVI-WSA dG” (60) considered as refinement method and scores, respectively. We have generated 30 poses for the placement method and five poses for the refinement method. The third docking process was carried out by using “Induced Fit” protocol. In this step, the protein was made flexible to fit the conformation with the substrate. The rest of the parameters under the current docking run remain the same as the previous docking simulation. At the end of the simulation, we chose the best substrate pose score according to their Gibbs free binding energy (ΔG binding), RMSD, and binding affinity between substrate and the protein models.

Depending on the settings used, the final result database contains the following fields: the final score (**S**); the RMSD between the pose and the original substrate or between the poses before and after refinement (rmsd and rmsd_refine, Å); energy of conformer (E_conf); and scores for successive docking stages: placement, rescoring, and refinement (E_place, E_score1, E_score2, and E_refine). Lower final S-scores indicate more favorable poses. The detailed docking results of protein models are provided in **Table 1**.

### Molecular dynamics and simulation

MD simulation was carried out to understand the stability and dynamic behavior of modeled protein–substrate docked complexes. We used Desmond with Optimized Potentials for Liquid Simulations (OPLS4) as force field (Desmond, version 4.7., Schrodinger, LLC, New York, NY, 2022) in the experiments. The protein–substrate was saturated with Simple Point Charge (SPC) water model as solvent inside an orthorhombic box. It is further neutralized by adding appropriate counter ions and 0.15 M salt concentration (61). The distance between the protein–substrate and box wall was set to 10 Å to avoid the steric interaction. It was further minimized by applying a hybrid method of steepest descent and the Limited Memory Broyden–Fletcher–Goldfarb–Shanno algorithms for 100 ps until a gradient threshold of 25 kcal/mol is achieved (62). The temperature was maintained at 300 K for whole simulations using Nose–Hoover thermostats. The Martyna–Tobias–Klein barostat method was used to maintain stable pressure. We performed NPT ensemble for the equilibrated system for 100 ns. To examine the equation of motion in dynamics, a multi-time step Reference System propagation algorithms (RESPA) integrator algorithm was used (63). The final equilibrated system is used to perform a 100-ns molecular dynamics simulation run. The systems were relaxed by constant NVT (number of particles, volume, and temperature) ensemble conditions for 1 ns to produce simulation data for post-simulation analysis (64, 65) The results were analyzed through simulation interaction diagram and trajectory plot module of Desmond.

### Binding free energy calculation

The binding free energies of the protein–substrate complexes were calculated through Prime-MM/GBSA (Prime, Schrodinger, LLC, New York, NY, 2022) to validate the IFD results. MM/GBSA combines procedure that integrates OPLS molecular mechanics energies (EMM), an Surface Generalized Born (SGB) model solvation model for polar solvation (GSGB), and a non-polar solvation term (GNP). It comprises of nonpolar solvent accessible surface area and van der Waals interactions (66). The binding free energy calculation is expressed as follows:


(1) ΔGbind=Gcomplex−(Gprotein+Gsubstrate)

where


(2) G=EMM+GSGB+GNP

The Gaussian surface area model was preferred over van der Waals by Prime (Prime, Schrodinger, LLC, New York, NY, 2022) for representing the solvent-accessible surface area (67).

### Substrate transport analysis

Caver Web 1.0 (68) is used to study the comprehensive analysis of protein tunnels and channels for the substrate transport. The identified tunnels, their properties, energy profiles, and trajectories for substrate passages can be calculated and visualized. The workflow involves four steps. The first step involves the selection of a protein structure and its pre-treatment. The second step is a selection of a starting point selection for tunnel detection. Protein tunnels are identified and analyzed in the third step. The final step involves the selected substrate transport and the energy profile analysis for the selected tunnels.

### Alanine scan methodology

Alanine scanning is a technique used to determine the importance of a residue to the stability, affinity, or property of a given protein. Alanine is used because its side chain is non-bulky and chemically inert while retaining secondary structure preferences like most amino acids (69).

We have provided the results in the form of Stability: It defines the absolute thermostability of the mutation. Stability is equal to the Boltzmann average of the stabilities of the ensemble. A more negative value indicates a more stable mutation. dStability: It defines the relative thermostability of the mutation with respect to the wild-type protein. dStability is the difference of the Boltzmann averages of the mutant and wild-type stabilities of the ensemble. A more negative value indicates more stable mutation. The units of stability are kcal/mol. Affinity: The absolute binding affinity of the mutation. Affinity is equal to the Boltzmann average of the affinities of the ensemble. A more negative value indicates a mutation with better affinity. The units of affinity are kcal/mol. dAffinity: The relative binding affinity of the mutation to the wild-type protein. dAffinity is the difference of the Boltzmann averages of the mutant and wild-type affinities of the ensemble. A more negative value indicates a mutation with better affinity. The units of affinity are kcal/mol. The settings incorporated are “Repack environment” and the “Cutoff” distance (Å) used to determine which residues are added to the conformational search in addition to the residue being mutated. Our Cutoff value is 4.5Å. We have selected the “Refined Mutations” option that allows mutations to be refined by following conformational space using UQO. “RMS Gradient” option allows final refinement until RMS of the gradient of the potential energy is less than a specified value.

## Data availability statement

The TMT LC-MS/MS proteomics data have been deposited to the ProteomeXchange Consortium via the PRIDE (70) partner repository with the dataset identifier PXD025574.

## Ethics statement

The animal study was approved by Sanger Institute’s research review boards. The study was conducted in accordance with the local legislation and institutional requirements.

## Author contributions

SoR designed the experiments. SoR performed the experiments. SuR did the molecular docking and simulation studies. BM and JP provided the murine Th2 cells when required. SR wrote the manuscript with help from SuR. MH did the on-bead digestion and mass spectrometry of CD4^+^ Th2 cells. ST and A-CG supervised the study. All authors commented on and approved the draft manuscript before submission.

## References

[1] Shih M-CM Chiu Y-N Hu M-C Guo I-C Chung B. Regulation of steroid production: analysis of Cyp11a1 promoter. Mol Cell Endocrinol (2011) 336:80–4. doi: 10.1016/j.mce.2010.12.017 21195129

[2] Payne AH Hales DB. Overview of steroidogenic enzymes in the pathway from cholesterol to active steroid hormones. Endocr Rev (2004) 25:947–70. doi: 10.1210/er.2003-0030 15583024

[3] Legacki EL Ball BA Corbin CJ Loux SC Scoggin KE Stanley SD. Equine fetal adrenal, gonadal and placental steroidogenesis. Reproduction (2017) 154:445–54. doi: 10.1530/REP-17-0239 28878092

[4] Miller WL Auchus RJ. The molecular biology, biochemistry, and physiology of human steroidogenesis and its disorders. Endocr Rev (2011) 32:81–151. doi: 10.1210/er.2010-0013 21051590 PMC3365799

[5] Weng J-H Chung B. Nongenomic actions of neurosteroid pregnenolone and its metabolites. Steroids (2016) 111:54–9. doi: 10.1016/j.steroids.2016.01.017 26844377

[6] Mayo W Le Moal M Abrous DN. Pregnenolone sulfate and aging of cognitive functions: behavioral, neurochemical, and morphological investigations. Hormones Behav (2001) 40:215–7. doi: 10.1006/hbeh.2001.1677 11534985

[7] Mellon SH. Neurosteroid regulation of central nervous system development. Pharmacol Ther (2007) 116:107–24. doi: 10.1016/j.pharmthera.2007.04.011 PMC2386997 17651807

[8] Marx CE Lee J Subramaniam M Rapisarda A Bautista DCT Chan E. Proof-of-concept randomized controlled trial of pregnenolone in schizophrenia. Psychopharmacol (Berl) (2014) 231:3647–62. doi: 10.1007/s00213-014-3673-4 25030803

[9] Grigoryev DN Long BJ Njar VC Brodie AH. Pregnenolone stimulates LNCaP prostate cancer cell growth via the mutated androgen receptor. J Steroid Biochem Mol Biol (2000) 75:1–10. doi: 10.1016/S0960-0760(00)00131-X 11179903

[10] Trabert B Geczik AM Bauer DC Buist DSM Cauley JA Falk RT. Association of endogenous pregnenolone, progesterone, and related metabolites with risk of endometrial and ovarian cancers in postmenopausal women: the B~FIT cohort. Cancer Epidemiol Biomarkers Prev (2021) 30:2030–7. doi: 10.1158/1055-9965.EPI-21-0669 PMC8568650 34465588

[11] Xiao X Chen L Ouyang Y Zhu W Qiu P Su X. Pregnenolone, a cholesterol metabolite, induces glioma cell apoptosis via activating extrinsic and intrinsic apoptotic pathways. Oncol Lett (2014) 8:645–50. doi: 10.3892/ol.2014.2147 PMC4081362 25013479

[12] Murugan S Jakka P Namani S Mujumdar V Radhakrishnan G. The neurosteroid pregnenolone promotes degradation of key proteins in the innate immune signaling to suppress inflammation. J Biol Chem (2019) 294:4596–607. doi: 10.1074/jbc.RA118.005543 PMC6433066 30647133

[13] Weng J-H Liang M.-R Chen C.-H Tong S-K Huang T.-C Lee S-P. Pregnenolone activates CLIP-170 to promote microtubule growth and cell migration. Nat Chem Biol (2013) 9:636–42. doi: 10.1038/nchembio.1321 23955365

[14] Hamasaki M Matsumura S Satou A Takahashi C Oda Y Higashiura C. Pregnenolone functions in centriole cohesion during mitosis. Chem Biol (2014) 21:1707–21. doi: 10.1016/j.chembiol.2014.11.005 25525990

[15] Mahata B Zhang X Kolodziejczyk AA Proserpio V Haim-Vilmovsky L Taylor AE. Single-cell RNA sequencing reveals T helper cells synthesizing steroids *de novo* to contribute to immune homeostasis. Cell Rep (2014) 7:1130–42. doi: 10.1016/j.celrep.2014.04.011 PMC4039991 24813893

[16] Jia Y Domenico J Takeda K Han J Wang M Armstrong M. Steroidogenic enzyme Cyp11a1 regulates Type 2 CD8+ T cell skewing in allergic lung disease. PNAS (2013) 110:8152–7. doi: 10.1073/pnas.1216671110 PMC3657775 23630275

[17] Luckheeram RV Zhou R Verma AD Xia B. CD4+T cells: differentiation and functions. Clin Dev Immunol (2012) 2012:925135. doi: 10.1155/2012/925135 22474485 PMC3312336

[18] Kennedy R Celis E. Multiple roles for CD4+ T cells in anti-tumor immune responses. Immunol Rev (2008) 222:129–44. doi: 10.1111/j.1600-065X.2008.00616.x 18363998

[19] Chen F Liu Z Wu W Rozo C Bowdridge S Millman A. An essential role for the Th2-type response in limiting tissue damage during helminth infection. Nat Med (2012) 18:260–6. doi: 10.1038/nm.2628 PMC3274634 22245779

[20] Anthony RM Urban JF Alem F Hamed HA Rozo CT Boucher J-L. Memory T(H)2 cells induce alternatively activated macrophages to mediate protection against nematode parasites. Nat Med (2006) 12:955–60. doi: 10.1038/nm1451 PMC1955764 16892038

[21] Liu Q Kreider T Bowdridge S Liu Z Song Y Gaydo AG. B cells have distinct roles in host protection against different nematode parasites. J Immunol (2010) 184:5213–23. doi: 10.4049/jimmunol.0902879 PMC3729113 20357259

[22] Roy S Sipthorp J Mahata B Pramanik J Hennrich ML Gavin A-C. CLICK-enabled analogues reveal pregnenolone interactomes in cancer and immune cells. iScience (2021) 24:102485. doi: 10.1016/j.isci.2021.102485 34036248 PMC8138728

[23] Fu Y Zhao J Chen Z. Insights into the molecular mechanisms of protein-ligand interactions by molecular docking and molecular dynamics simulation: A case of oligopeptide binding protein. Comput Math Methods Med (2018) 2018:3502514. doi: 10.1155/2018/3502514 30627209 PMC6305025

[24] Du X Li Y Xia Y-L Ai S-M Liang J Sang P. Insights into protein–ligand interactions: mechanisms, models, and methods. Int J Mol Sci (2016) 17:144. doi: 10.3390/ijms17020144 26821017 PMC4783878

[25] Venkatachalam CM Jiang X Oldfield T Waldman M. LigandFit: a novel method for the shape-directed rapid docking of ligands to protein active sites. J Mol Graph Model (2003) 21:289–307. doi: 10.1016/S1093-3263(02)00164-X 12479928

[26] Osterberg F Morris GM Sanner MF Olson AJ Goodsell DS. Automated docking to multiple target structures: incorporation of protein mobility and structural water heterogeneity in AutoDock. Proteins (2002) 46:34–40. doi: 10.1002/prot.10028 11746701

[27] Rarey M Kramer B Lengauer T Klebe G. A fast flexible docking method using an incremental construction algorithm. J Mol Biol (1996) 261:470–89. doi: 10.1006/jmbi.1996.0477 8780787

[28] Jain AN. Surflex: Fully automatic flexible molecular docking using a molecular similarity-based search engine. J Med Chem (2003) 46:499–511. doi: 10.1021/jm020406h 12570372

[29] Jones G Willett P Glen RC Leach AR Taylor R. Development and validation of a genetic algorithm for flexible docking. J Mol Biol (1997) 267:727–48. doi: 10.1006/jmbi.1996.0897 9126849

[30] Schapira M Abagyan R Totrov M. Nuclear hormone receptor targeted virtual screening. J Med Chem (2003) 46:3045–59. doi: 10.1021/jm0300173 12825943

[31] Friesner RA Banks JL Murphy RB Halgren TA Klicic JJ Mainz DT. Glide: A new approach for rapid, accurate docking and scoring. 1. Method and assessment of docking accuracy. J Med Chem (2004) 47:1739–49. doi: 10.1021/jm0306430 15027865

[32] McGann MR Almond HR Nicholls A Grant JA Brown FK. Gaussian docking functions. Biopolymers (2003) 68:76–90. doi: 10.1002/bip.10207 12579581

[33] Corbeil CR Williams CI Labute P. Variability in docking success rates due to dataset preparation. J Comput Aided Mol Des (2012) 26:775–86. doi: 10.1007/s10822-012-9570-1 PMC3397132 22566074

[34] Zhao H Caflisch A. Discovery of ZAP70 inhibitors by high-throughput docking into a conformation of its kinase domain generated by molecular dynamics. Bioorg Med Chem Lett (2013) 23:5721–6. doi: 10.1016/j.bmcl.2013.08.009 23993776

[35] Trott O Olson AJ. AutoDock Vina: improving the speed and accuracy of docking with a new scoring function, efficient optimization and multithreading. J Comput Chem (2010) 31:455–61. doi: 10.1002/jcc.21334 PMC3041641 19499576

[36] Ruiz-Carmona S Alvarez-Garcia D Foloppe N Garmendia-Doval AB Juhos S Schmidtke P. rDock: A fast, versatile and open source program for docking ligands to proteins and nucleic acids. PloS Comput Biol (2014) 10:e1003571. doi: 10.1371/journal.pcbi.1003571 24722481 PMC3983074

[37] Allen WJ Balius TE Mukherjee S Brozell SR Moustakas DT Lang PT. DOCK 6: impact of new features and current docking performance. J Comput Chem (2015) 36:1132–56. doi: 10.1002/jcc.23905 PMC4469538 25914306

[38] Vallée M Vitiello S Bellocchio L Hébert-Chatelain E Monlezun S Martin-Garcia E. Pregnenolone can protect the brain from cannabis intoxication. Science (2014) 343:94–8. doi: 10.1126/science.1243985 PMC4057431 24385629

[39] Murakami K Fellous A Baulieu EE Robel P. Pregnenolone binds to microtubule-associated protein 2 and stimulates microtubule assembly. Proc Natl Acad Sci U.S.A. (2000) 97:3579–84. doi: 10.1073/pnas.97.7.3579 PMC16282 10737804

[40] Kleiner P Heydenreuter W Stahl M Korotkov VS. & Sieber, S. A. A whole proteome inventory of background photocrosslinker binding. Angewandte Chemie Int Edition (2017) 56:1396–401. doi: 10.1002/anie.201605993 27981680

[41] Mzhavia N Berman YL Qian Y Yan L Devi LA. Cloning, expression, and characterization of human metalloprotease 1: a novel member of the pitrilysin family of metalloendoproteases. DNA Cell Biol (1999) 18:369–80. doi: 10.1089/104454999315268 10360838

[42] Tonazzi A Giangregorio N Console L Palmieri F Indiveri C. The mitochondrial carnitine acyl-carnitine carrier (SLC25A20): molecular mechanisms of transport, role in redox sensing and interaction with drugs. Biomolecules (2021) 11:521. doi: 10.3390/biom11040521 33807231 PMC8066319

[43] Mastorodemos V Kotzamani D Zaganas I Arianoglou G Latsoudis H Plaitakis A. Human GLUD1 and GLUD2 glutamate dehydrogenase localize to mitochondria and endoplasmic reticulum. Biochem Cell Biol (2009) 87:505–16. doi: 10.1139/O09-008 19448744

[44] Mahata B Pramanik J van der Weyden L Polanski K Kar G Riedel A. Tumors induce *de novo* steroid biosynthesis in T cells to evade immunity. Nat Commun (2020) 11:3588. doi: 10.1038/s41467-020-17339-6 32680985 PMC7368057

[45] Angajala A Lim S Phillips JB Kim J-H Yates C You Z. Diverse roles of mitochondria in immune responses: novel insights into immuno-metabolism. Front Immunol (2018) 9:1605. doi: 10.3389/fimmu.2018.01605 30050539 PMC6052888

[46] Jiang Y Tao Z Chen H Xia S. Endoplasmic reticulum quality control in immune cells. Front Cell Dev Biol (2021) 9. doi: 10.3389/fcell.2021.740653 PMC8511527 34660599

[47] Gao J Schatton D Martinelli P Hansen H Pla-Martin D Barth E. CLUH regulates mitochondrial biogenesis by binding mRNAs of nuclear-encoded mitochondrial proteins. J Cell Biol (2014) 207:213–23. doi: 10.1083/jcb.201403129 PMC4210445 25349259

[48] Yang C Ko B Hensley CT Jiang L Wasti AT Kim J. Glutamine oxidation maintains the TCA cycle and cell survival during impaired mitochondrial pyruvate transport. Mol Cell (2014) 56:414–24. doi: 10.1016/j.molcel.2014.09.025 PMC4268166 25458842

[49] Matias MI Yong CS Foroushani A Goldsmith C Mongellaz C Sezgin E. Regulatory T cell differentiation is controlled by αKG-induced alterations in mitochondrial metabolism and lipid homeostasis. Cell Rep (2021) 37:109911. doi: 10.1016/j.celrep.2021.109911 34731632 PMC10167917

[50] Smith HQ Li C Stanley CA Smith TJ. Glutamate dehydrogenase, a complex enzyme at a crucial metabolic branch point. Neurochem Res (2019) 44:117–32. doi: 10.1007/s11064-017-2428-0 PMC5924581 29079932

[51] Michaelis EK Wang X Pal R Bao X Hascup KN Wang Y. Neuronal glud1 (Glutamate dehydrogenase 1) over-expressing mice: increased glutamate formation and synaptic release, loss of synaptic activity, and adaptive changes in genomic expression. Neurochem Int (2011) 59:473–81. doi: 10.1016/j.neuint.2011.03.003 PMC3152645 21397652

[52] Chakraborty S Pramanik J Mahata B. Revisiting steroidogenesis and its role in immune regulation with the advanced tools and technologies. Genes Immun (2021) 22:125–40. doi: 10.1038/s41435-021-00139-3 PMC8277576 34127827

[53] Hughes CS Foehr S Garfield DA Furlong EE Steinmetz LM Krijgsveld J. Ultrasensitive proteome analysis using paramagnetic bead technology. Mol Syst Biol (2014) 10:757. doi: 10.15252/msb.20145625 25358341 PMC4299378

[54] Franken H Mathieson T Childs D Sweetman GMA Werner T Tögel I. Thermal proteome profiling for unbiased identification of direct and indirect drug targets using multiplexed quantitative mass spectrometry. Nat Protoc (2015) 10:1567–93. doi: 10.1038/nprot.2015.101 26379230

[55] Ritchie ME Phipson B Wu D Hu Y Law CW Shi W. limma powers differential expression analyses for RNA-sequencing and microarray studies. Nucleic Acids Res (2015) 43:e47. doi: 10.1093/nar/gkv007 25605792 PMC4402510

[56] Huber W von Heydebreck A Sültmann H Poustka A Vingron M. Variance stabilization applied to microarray data calibration and to the quantification of differential expression. Bioinformatics (2002) 18:S96–S104. doi: 10.1093/bioinformatics/18.suppl_1.S96 12169536

[57] Strimmer K. fdrtool: a versatile R package for estimating local and tail area-based false discovery rates. Bioinformatics (2008) 24:1461–2. doi: 10.1093/bioinformatics/btn209 18441000

[58] Jumper J Evans R Pritzel A Green T Figurnov M Ronneberger O. Highly accurate protein structure prediction with AlphaFold. Nature (2021) 596:583–9. doi: 10.1038/s41586-021-03819-2 PMC8371605 34265844

[59] Varadi M Anyango S Deshpande M Nair S Natassia C Yordanova G. AlphaFold Protein Structure Database: massively expanding the structural coverage of protein-sequence space with high-accuracy models. Nucleic Acids Res (2022) 50:D439–44. doi: 10.1093/nar/gkab1061 PMC8728224 34791371

[60] Labute P. The generalized Born/volume integral implicit solvent model: estimation of the free energy of hydration using London dispersion instead of atomic surface area. J Comput Chem (2008) 29:1693–8. doi: 10.1002/jcc.20933 18307169

[61] Das M Prakash S Nayak C Thangavel N Singh SK Manisankar P. Dihydroactinidiolide, a natural product against Aβ25-35 induced toxicity in Neuro2a cells: Synthesis, in silico and in *vitro* studies. Bioorg Chem (2018) 81:340–9. doi: 10.1016/j.bioorg.2018.08.037 30189414

[62] Vijayalakshmi P Selvaraj C Singh SK Nisha J Saipriya K Daisy P. Exploration of the binding of DNA binding ligands to Staphylococcal DNA through QM/MM docking and molecular dynamics simulation. J Biomol Struct Dyn (2013) 31:561–71. doi: 10.1080/07391102.2012.706080 22881193

[63] Patidar K Deshmukh A Bandaru S Lakkaraju C Girdhar A Vr G. Virtual screening approaches in identification of bioactive compounds akin to delphinidin as potential HER2 inhibitors for the treatment of breast cancer. Asian Pac J Cancer Prev (2016) 17:2291–5. doi: 10.7314/APJCP.2016.17.4.2291 27221932

[64] Suryanarayanan V Singh SK. Assessment of dual inhibition property of newly discovered inhibitors against PCAF and GCN5 through in silico screening, molecular dynamics simulation and DFT approach. J Recept Signal Transduct Res (2015) 35:370–80. doi: 10.3109/10799893.2014.956756 25404235

[65] Reddy KK Singh SK Tripathi SK Selvaraj C. Identification of potential HIV-1 integrase strand transfer inhibitors: in silico virtual screening and QM/MM docking studies. SAR QSAR Environ Res (2013) 24:581–95. doi: 10.1080/1062936X.2013.772919 23521430

[66] Salgado-Moran G Ramirez-Tagle R Glossman-Mitnik D Ruiz-Nieto S Kishore-Deb P Bunster M. Docking studies of binding of ethambutol to the C-terminal domain of the arabinosyltransferase from *Mycobacterium tuberculosis* . J Chem (2013) 2013:e601270. doi: 10.1155/2013/601270

[67] Pradiba D Aarthy M Shunmugapriya V Singh SK Vasanthi M. Structural insights into the binding mode of flavonols with the active site of matrix metalloproteinase-9 through molecular docking and molecular dynamic simulations studies. J Biomol Struct Dyn (2018) 36:3718–39. doi: 10.1080/07391102.2017.1397058 29068268

[68] Stourac J Vavra O Kokkonen P Filipovic J Pinto G Brezovsky J. Caver Web 1.0: identification of tunnels and channels in proteins and analysis of ligand transport. Nucleic Acids Res (2019) 47:W414–22. doi: 10.1093/nar/gkz378 PMC6602463 31114897

[69] Dehouck Y Grosfils A Folch B Gilis D Bogaerts P Rooman M. Fast and accurate predictions of protein stability changes upon mutations using statistical potentials and neural networks: PoPMuSiC-2.0. Bioinformatics (2009) 25:2537–43. doi: 10.1093/bioinformatics/btp445 19654118

[70] Perez-Riverol Y Csordas A Bai J Bernal-Llinares M Hewapathirana S Kundu DJ. The PRIDE database and related tools and resources in 2019: improving support for quantification data. Nucleic Acids Res (2019) 47:D442–50. doi: 10.1093/nar/gky1106 PMC6323896 30395289

