# Supplementary material for: CLICK-chemoproteomics and molecular dynamics simulation reveals pregnenolone targets and their binding conformations in Th2 cells
Source: Front Immunol. 2023 Oct 31;14:1229703. doi: 10.3389/fimmu.2023.1229703 (PMC10644475; doi:10.3389/fimmu.2023.1229703)
Supplement: Supplementary file 1 [file DataSheet_1.pdf]

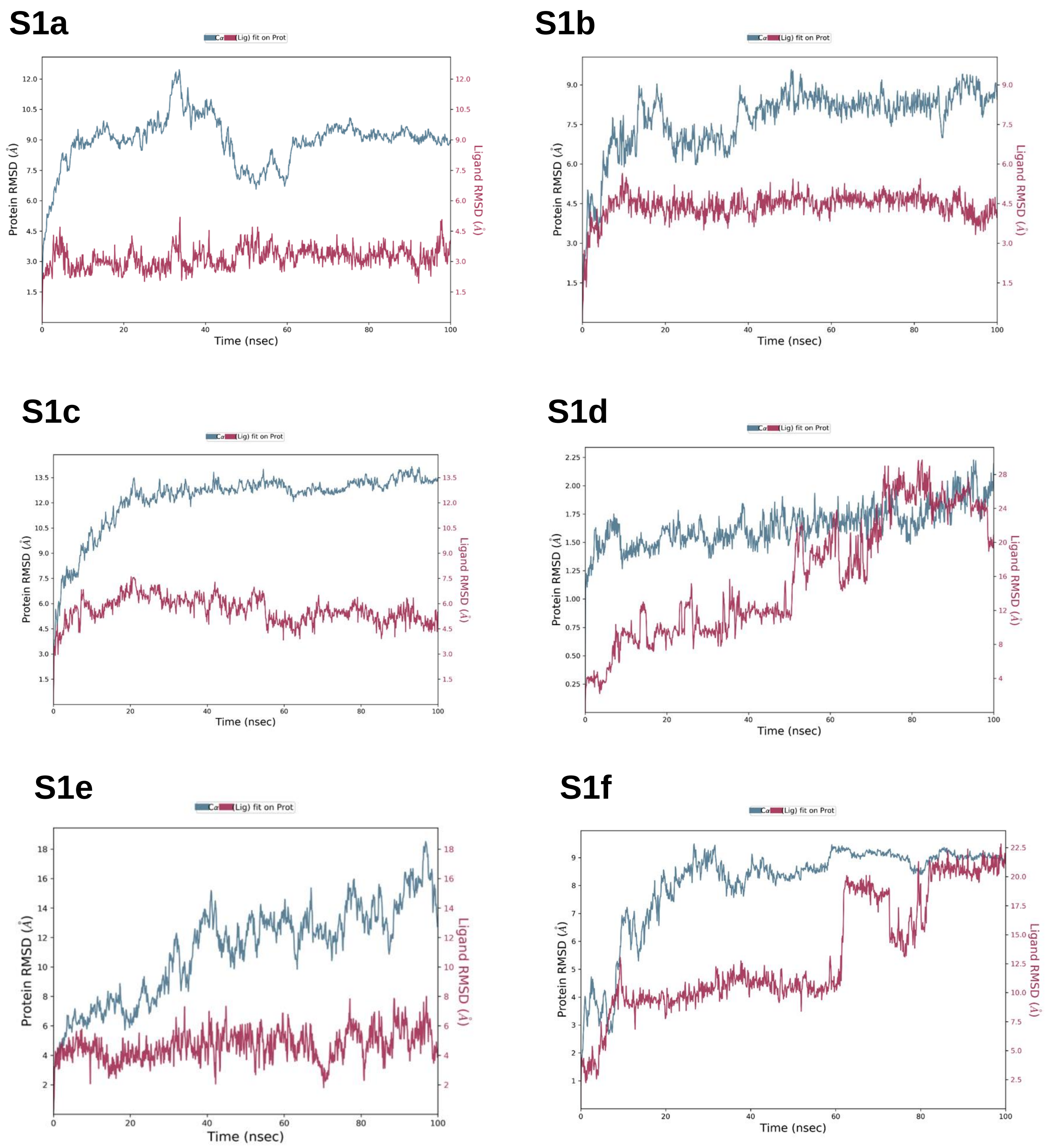

Figure S1. Protein substrate RMSD (a) P5-CLUH (b) P5-CYP51A1 (c) P5-GLUD1 (d) P5-LSS. (e) P5-P4HB. (f) P5-PITRM1

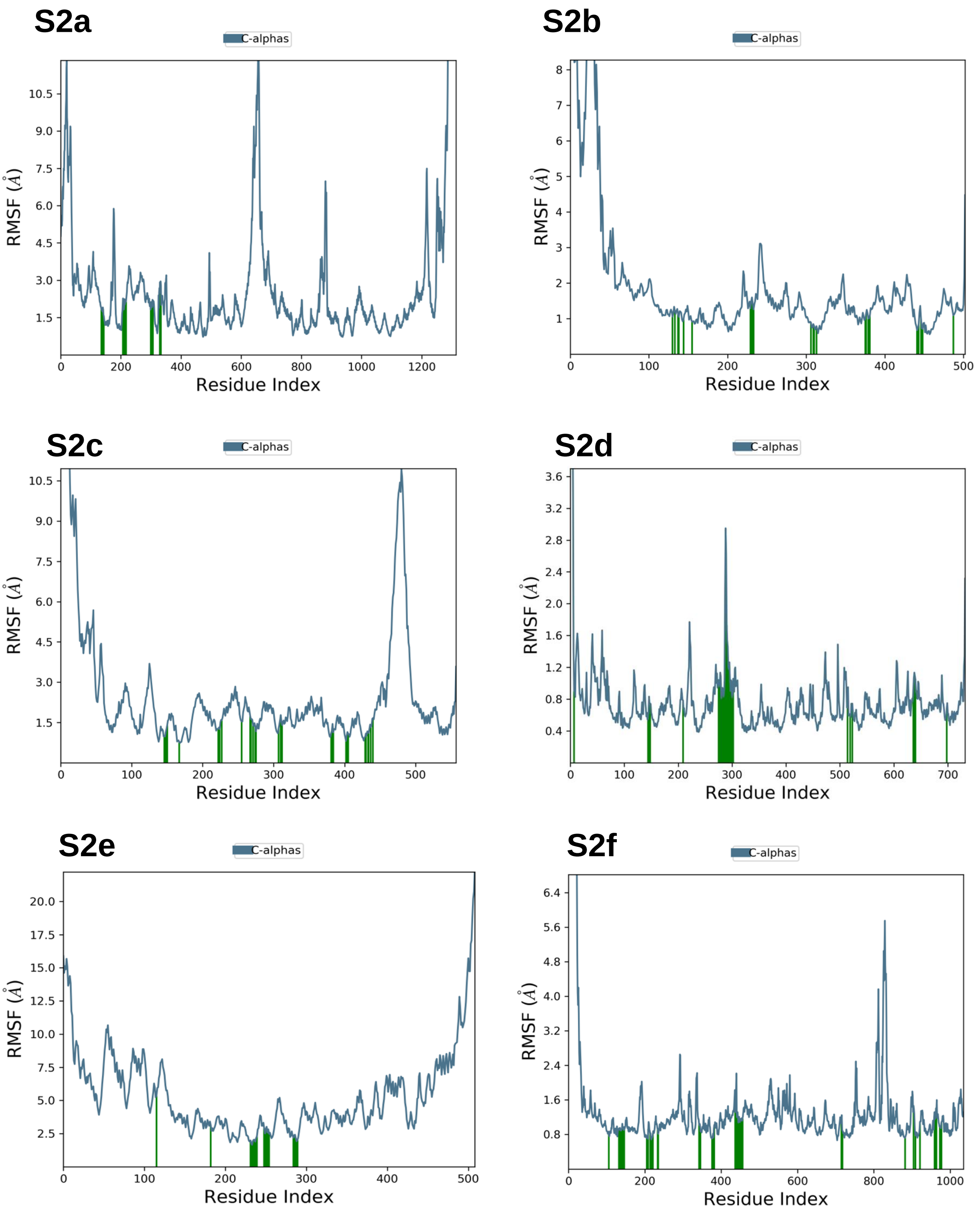

Figure S2. Protein RMSF (a) CLUH (b) CYP51A1 (c) GLUD1 (d) LSS. (e) P4HB. (f) PITRM1

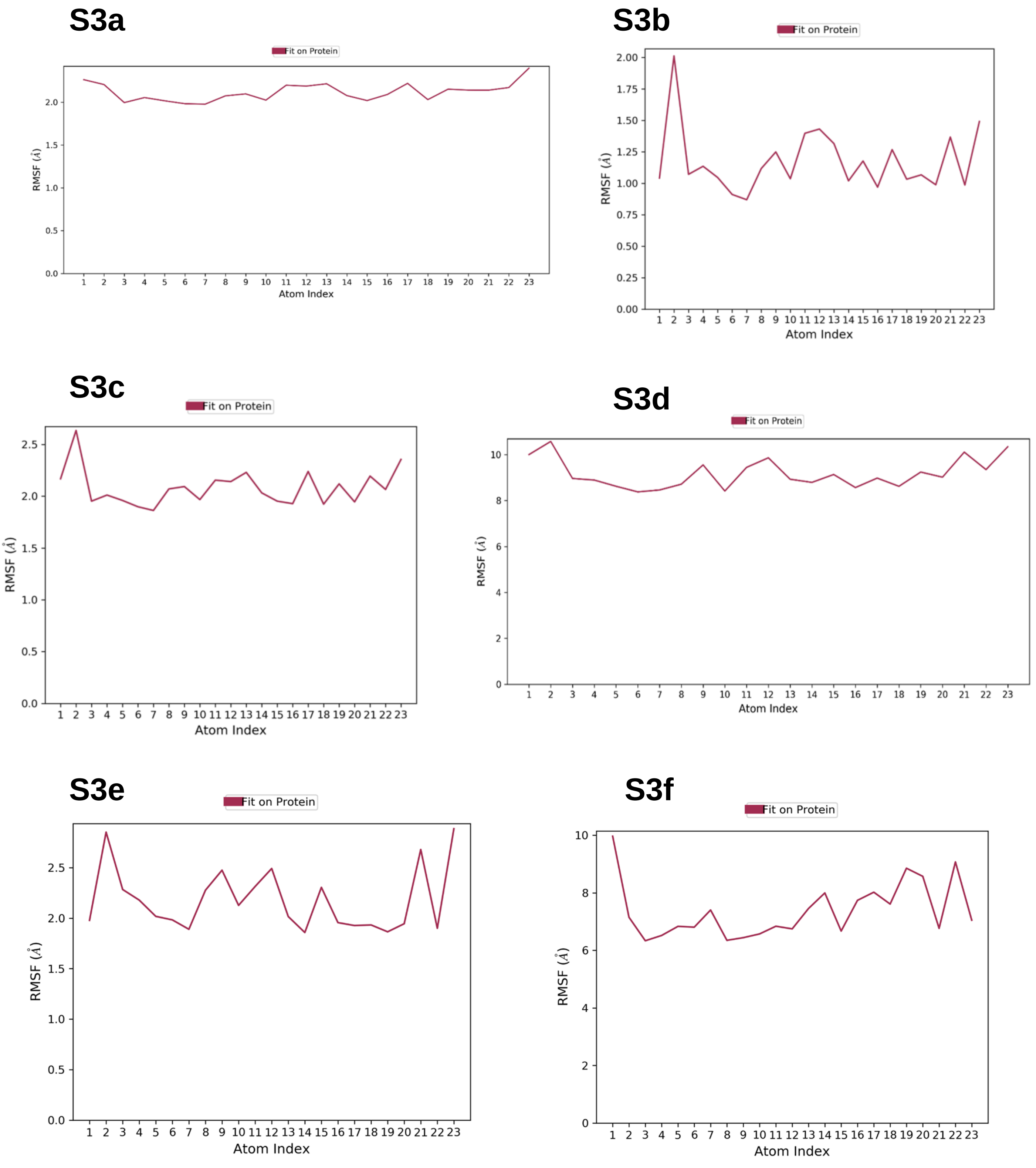

Figure S3. Substrate (P5) RMSF (a) CLUH (b) CYP51A1 (c) GLUD1 (d) LSS. (e) P4HB. (f) PITRM1

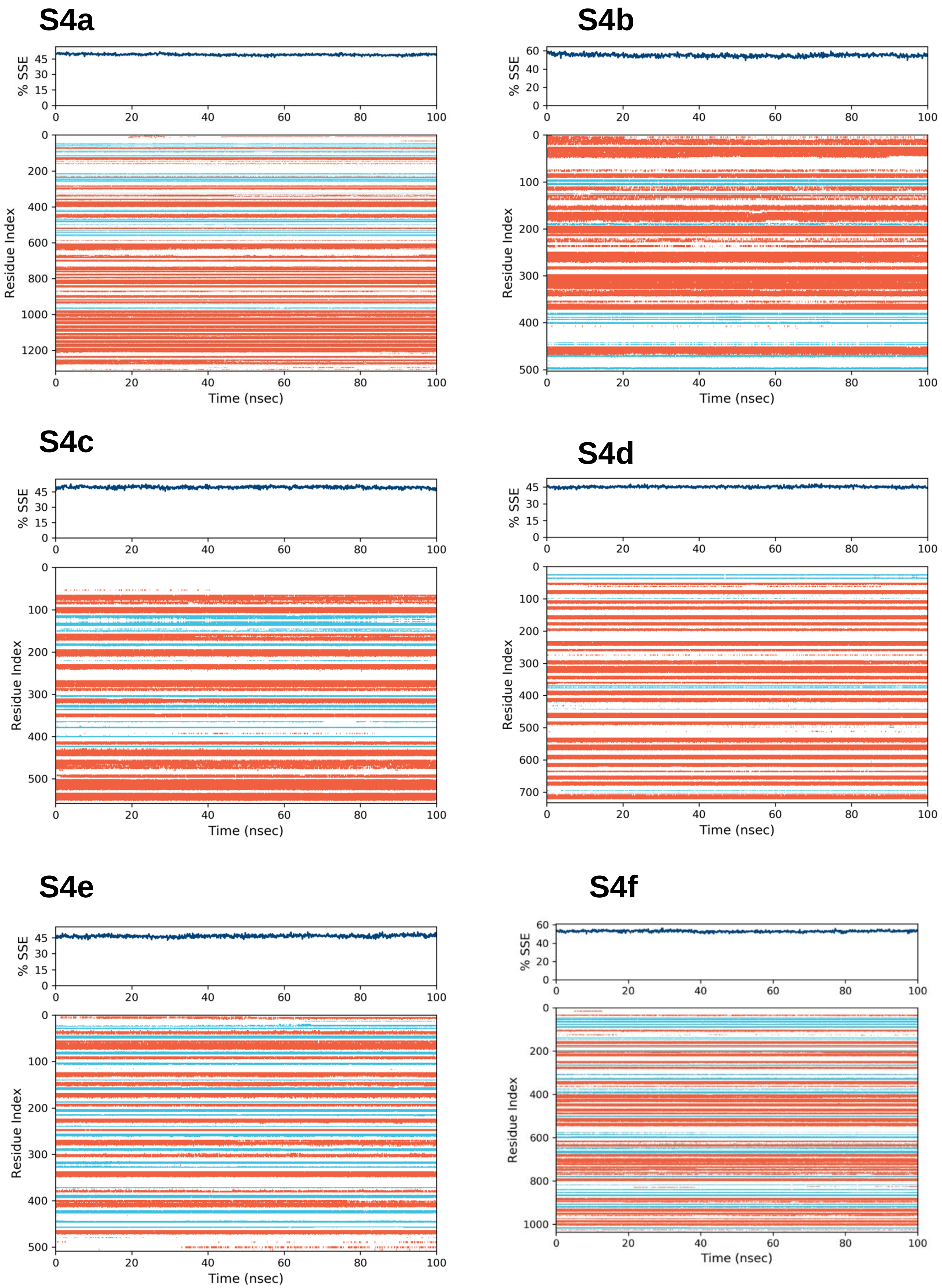

Figure S4. Secondary structure elements [SSE] (a) CLUH (b) CYP51A1 (c) GLUD1 (d) LSS. (e) P4HB. (f) PITRM1

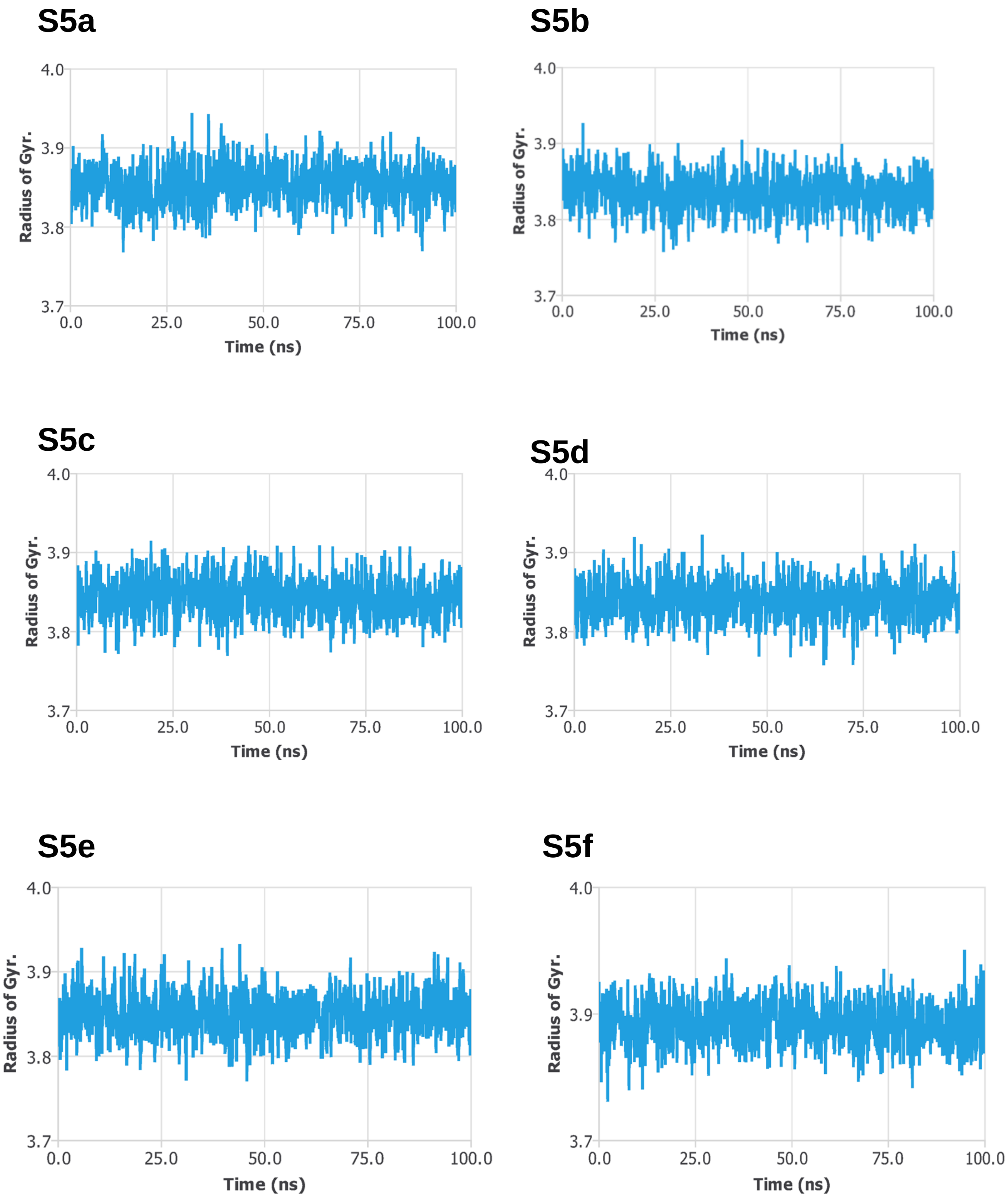

Figure S5. Radius of gyration (a) CLUH (b) CYP51A1 (c) GLUD1 (d) LSS. (e) P4HB. (f) PITRM1

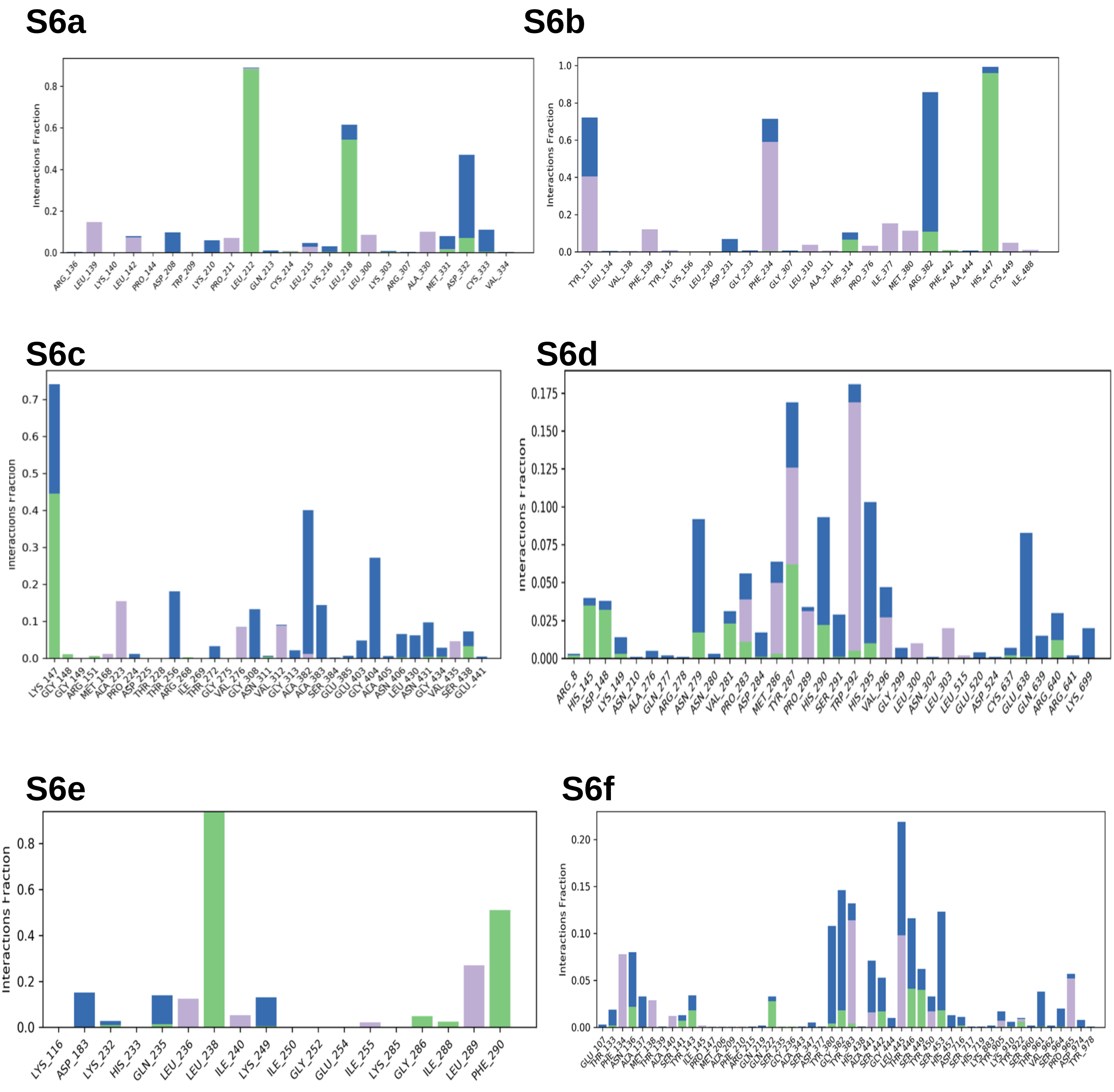

Figure S6. Protein - P5 contacts (a) CLUH (b) CYP51A1 (c) GLUD1 (d) LSS. (e) P4HB. (f) PITRM1

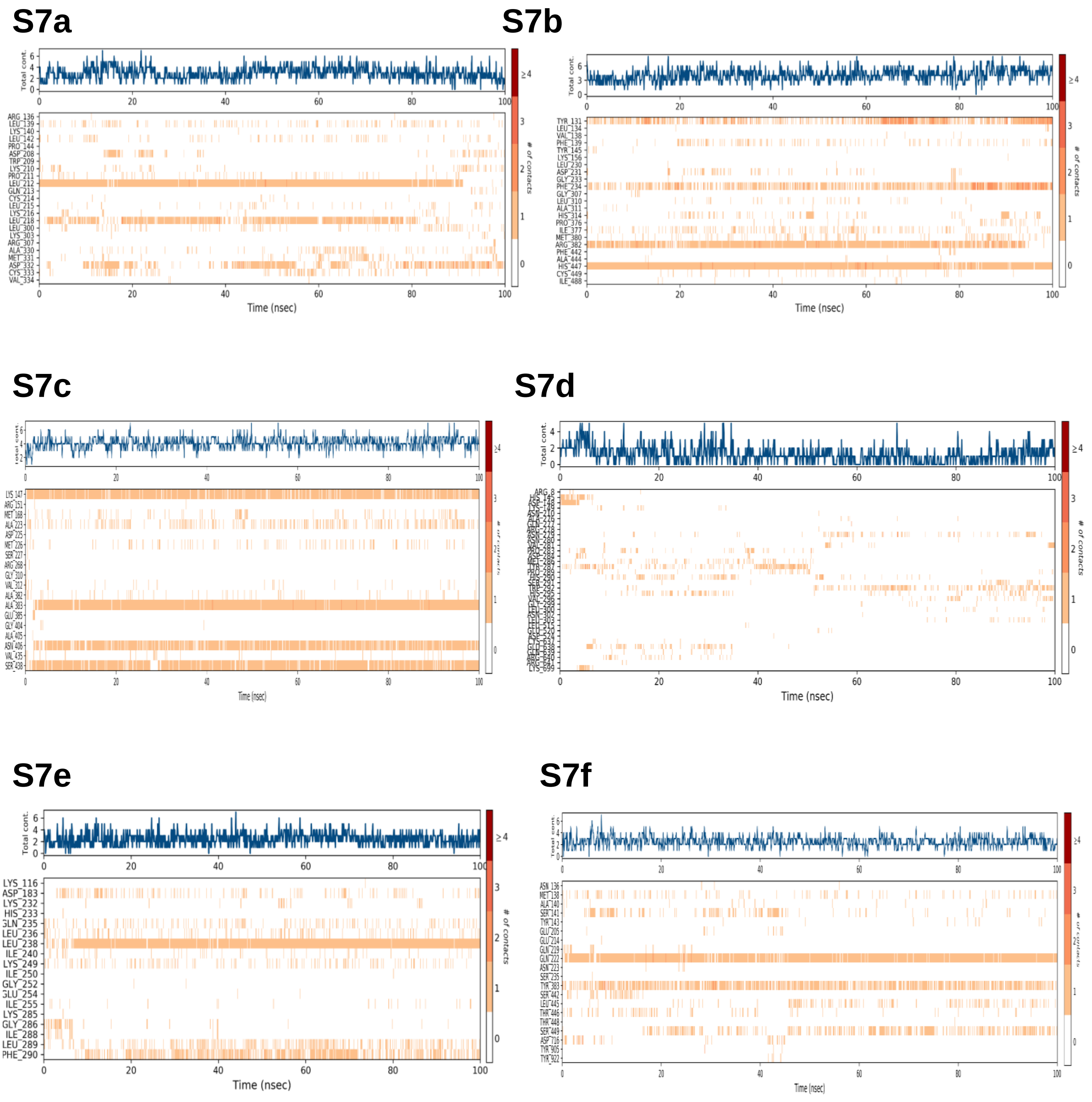

Figure S7. Protein - P5 timeline (a) CLUH (b) CYP51A1 (c) GLUD1 (d) LSS. (e) P4HB. (f) PITRM1

S8a

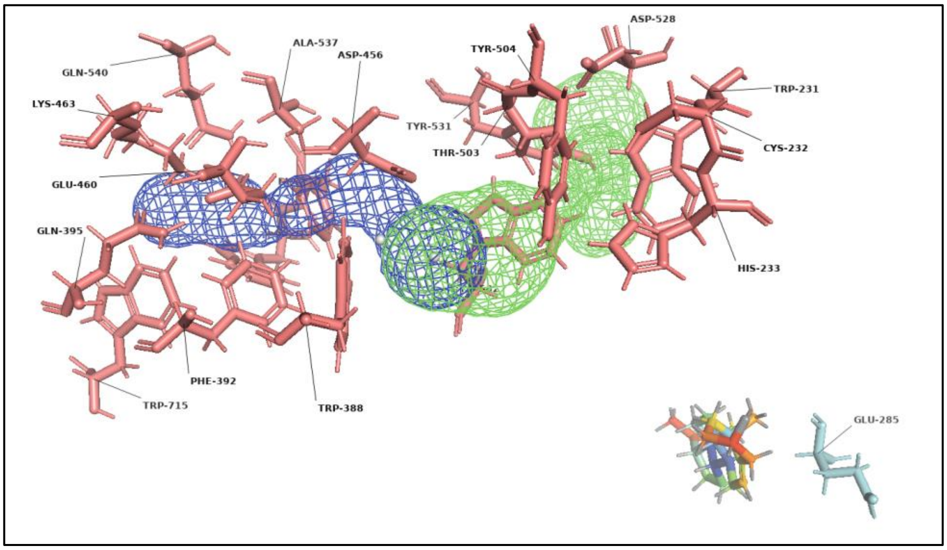

S8b

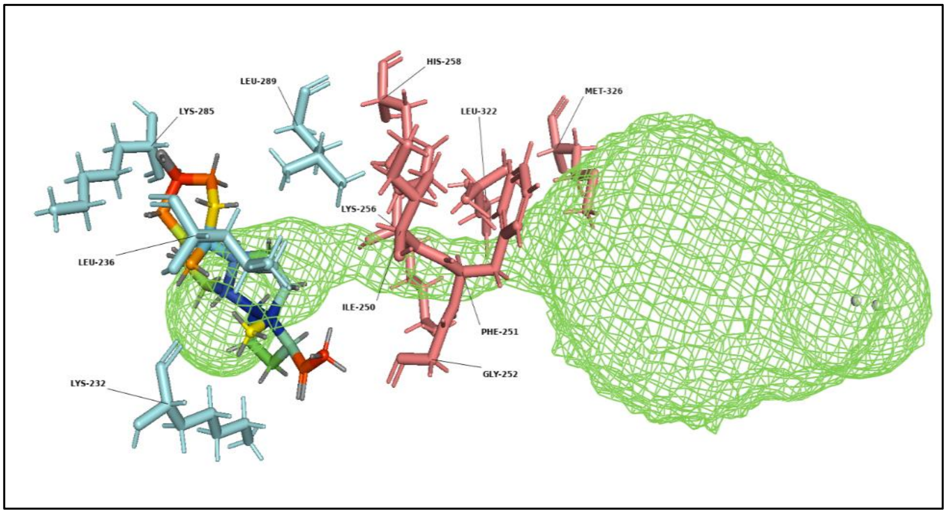

S8c

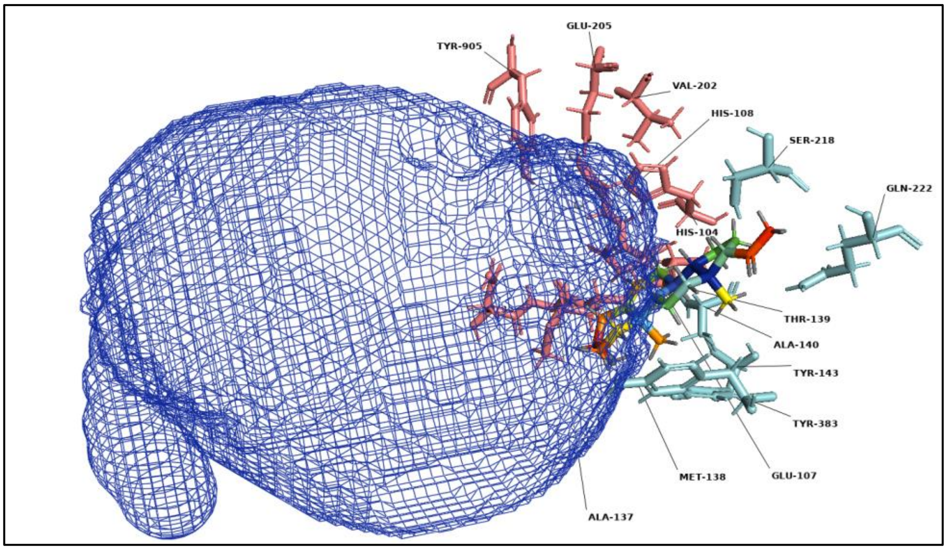

Figure S8. Non-involvement of tunnels for P5 transport. (a) LSS protein, Cyan color denotes hydrogen and hydrophobic interactions; Red Salmon color denotes other amino acids surrounding the tunnel. (b) P4HB protein, Cyan color denotes hydrogen and hydrophobic interactions; Red Salmon color denotes other amino acids surrounding the tunnel. (C) PITRM1 protein, Cyan color denotes hydrogen and hydrophobic interactions; Red Salmon color denotes other amino acids surrounding the tunnel. (b)
