# Supplementary material for: CLICK-chemoproteomics and molecular dynamics simulation reveals pregnenolone targets and their binding conformations in Th2 cells
Source: Front Immunol. 2023 Oct 31;14:1229703. doi: 10.3389/fimmu.2023.1229703 (PMC10644475; doi:10.3389/fimmu.2023.1229703)
Supplement: Supplementary file 2 [file DataSheet_2.docx]

**Protein-Ligand Interaction Profiler**

**Table ST1(a):** PLIF results of CLUH

| Proteins | **Hydrophobic interactions** |  |  |  |  | |  | | |  |  |  |
| --- | --- | --- | --- | --- | --- | --- | --- | --- | --- | --- | --- | --- |
|  | Index | Residue | AA | Distance | Substrate Atom | | Protein Atom | | |  |  |  |
| **CLUH** | 1 | 143A | ASP | 3.91 | 20723 | | 2213 | | |  |  |  |
|  | 2 | 332A | ASP | 3.80 | 20728 | | 5204 | | |  |  |  |
|  |  |  |  |  |  | |  | | |  |  |  |
| Proteins | **Hydrogen Bonds** |  |  |  |  |  | |  |  | |  |  |
|  | Index | Residues | AA | Distance H-A | Distance D-A | Donor Angle | | Protein Donor | Side Chain | | Donor Atom | Acceptor Atom |
| **CLUH** | 1 | 212A | LEU | 2.42 | 2.96 | 113.15 | | YES | NO | | 3253[Nam] | 20713[O2] |
|  | 2 | 218A | LEU | 2.17 | 2.99 | 137.32 | | YES | NO | | 3357[Nam] | 20712[O3] |

**Table ST1(b):** PLIF results of CYP51A1

| Proteins | **Hydrophobic interactions** | |  | |  | |  | |  | |  | |  |  |  |
| --- | --- | --- | --- | --- | --- | --- | --- | --- | --- | --- | --- | --- | --- | --- | --- |
|  | Index | | Residue | | AA | | Distance | | Substrate Atom | | Protein Atom | |  |  |  |
| **CYP51A1** | 1 | | 131A | | TYR | | 3.70 | | 8055 | | 1989 | |  |  |  |
|  | 2 | | 131A | | TYR | | 3.65 | | 8054 | | 1991 | |  |  |  |
|  | 3 | | 131A | | TYR | | 3.67 | | 8056 | | 1992 | |  |  |  |
|  | 4 | | 145A | | TYR | | 3.59 | | 8055 | | 2203 | |  |  |  |
|  | 5 | | 234A | | PHE | | 3.49 | | 8052 | | 3629 | |  |  |  |
|  | 6 | | 234A | | PHE | | 3.54 | | 8060 | | 3630 | |  |  |  |
|  | 7 | | 377A | | ILE | | 3.72 | | 8054 | | 5982 | |  |  |  |
|  | 8 | | 377A | | ILE | | 3.92 | | 8043 | | 5983 | |  |  |  |
|  | 9 | | 487A | | MET | | 3.86 | | 8049 | | 7732 | |  |  |  |
|  | 10 | | 488A | | ILE | | 3.78 | | 8049 | | 7753 | |  |  |  |
|  |  | |  | |  | |  | |  | |  | |  |  |  |
| Proteins | **Hydrogen Bonds** |  | |  | |  | |  | |  |  |  | |  |  |
|  | Index | Residue | | AA | | Distance H-A | | Distance D-A | | Donor Angle | Protein Donor | Side Chain | | Donor Atom | Acceptor Atom |
| **CYP51A1** | 1 | 145A | | TYR | | 3.28 | | 3.64 | | 104.61 | YES | YES | | 2204[O3] | 8038[O3] |
|  | 2 | 145A | | TYR | | 2.73 | | 3.64 | | 160.21 | NO | YES | | 8038[O3] | 2204[O3] |
|  | 3 | 231A | | ASP | | 3.21 | | 3.51 | | 101.27 | YES | YES | | 3601[O3] | 8039[O2] |

**Table ST1(c):** PLIF results of GLUD1

| Proteins | | **Hydrophobic interactions** | |  | |  | |  | |  | | |  | |  |  |  |
| --- | --- | --- | --- | --- | --- | --- | --- | --- | --- | --- | --- | --- | --- | --- | --- | --- | --- |
|  | | Index | | Residue | | AA | | Distance | | Substrate Atom | | | Protein Atom | |  |  |  |
| **GLUD1** | | 1 | | 311A | | ASN | | 3.87 | | 8614 | | | 4726 | |  |  |  |
|  | |  | |  | |  | |  | |  | | |  | |  |  |  |
| Proteins | **Hydrogen Bonds** | |  | |  | |  | |  | |  |  | |  | |  |  |
|  | Index | | Residue | | AA | | Distance H-A | | Distance D-A | | Donor Angle | Protein Donor | | Side Chain | | Donor Atom | Acceptor Atom |
| **GLUD1** | 1 | | 268A | | ARG | | 3.06 | | 3.79 | | 130.07 | YES | | YES | | 4094[Ng+] | 8605[O3] |
|  | 2 | | 268A | | ARG | | 2.23 | | 3.17 | | 153.56 | YES | | YES | | 4095[Ng+] | 8605[O3] |
|  | 3 | | 272A | | THR | | 2.88 | | 3.41 | | 115.63 | YES | | YES | | 4156[O3] | 8605[O3] |
|  | 4 | | 272A | | THR | | 2.93 | | 3.41 | | 112.11 | NO | | YES | | 8605[O3] | 4156[O3] |

**Table ST1(d):** PLIF results of LSS

| Proteins | | **Hydrophobic interactions** | |  | |  | |  | |  | |  | |  |  |  |
| --- | --- | --- | --- | --- | --- | --- | --- | --- | --- | --- | --- | --- | --- | --- | --- | --- |
|  | | Index | | Residue | | AA | | Distance | | Substrate Atom | | Protein Atom | |  |  |  |
| **LSS** | | 1 | | 285A | | GLU | | 3.96 | | 11505 | | 4472 | |  |  |  |
|  | |  | |  | |  | |  | |  | |  | |  |  |  |
| Proteins | **Hydrogen Bonds** | |  | |  | |  | |  |  |  | |  | |  |  |
|  | Index | | Residue | | AA | | Distance H-A | | Distance D-A | Donor Angle | Protein Donor | | Side Chain | | Donor Atom | Acceptor Atom |
| **LSS** | 1 | | 285A | | GLU | | 3.29 | | 3.78 | 114.36 | NO | | NO | | 11487[O3] | 4473[O2] |

**Table ST1(e):** PLIF results of P4HB

| Proteins | **Hydrophobic interactions** |  |  |  |  |  |
| --- | --- | --- | --- | --- | --- | --- |
|  | Index | Residue | AA | Distance | Substrate Atom | Protein Atom |
| **P4HB** | 1 | 232A | LYS | 3.57 | 7984 | 3557 |
|  | 2 | 236A | LEU | 3.37 | 7980 | 3628 |
|  | 3 | 285A | LYS | 3.36 | 7991 | 4409 |
|  | 4 | 289A | LEU | 3.49 | 7992 | 4480 |

**Table ST1(f):** PLIF results of PITRM1

| Proteins | | **Hydrophobic interactions** | |  | |  | |  | |  | | |  | |  |  |  |
| --- | --- | --- | --- | --- | --- | --- | --- | --- | --- | --- | --- | --- | --- | --- | --- | --- | --- |
|  | | Index | | Residue | | AA | | Distance | | Substrate Atom | | | Protein Atom | |  |  |  |
| **PITRM1** | | 1 | | 140A | | ALA | | 3.86 | | 16455 | | | 2246 | |  |  |  |
|  | | 2 | | 222A | | GLN | | 3.64 | | 16460 | | | 3584 | |  |  |  |
|  | | 3 | | 383A | | TYR | | 3.79 | | 16452 | | | 6069 | |  |  |  |
|  | | 4 | | 383A | | TYR | | 3.65 | | 16447 | | | 6073 | |  |  |  |
|  | | 5 | | 383A | | TYR | | 3.84 | | 16453 | | | 6074 | |  |  |  |
|  | | 6 | | 383A | | TYR | | 3.50 | | 16455 | | | 6072 | |  |  |  |
|  | |  | |  | |  | |  | |  | | |  | |  |  |  |
| Proteins | **Hydrogen Bonds** | |  | |  | |  | |  | |  |  | |  | |  |  |
|  | Index | | Residue | | AA | | Distance H-A | | Distance D-A | | Donor Angle | Protein Donor | | Side Chain | | Donor Atom | Acceptor Atom |
| **PITRM1** | 1 | | 143A | | TYR | | 2.90 | | 3.73 | | 145.47 | YES | | YES | | 2286[O3] | 16438[O3] |
|  | 2 | | 143A | | TYR | | 3.15 | | 3.73 | | 120.23 | NO | | YES | | 16438[O3] | 2286[O3] |
|  | 3 | | 218A | | SER | | 3.70 | | 4.03 | | 102.98 | YES | | YES | | 3522[O3] | 16439[O2] |
|  | 4 | | 222A | | GLN | | 2.68 | | 3.12 | | 106.49 | YES | | YES | | 3588[Nam] | 16439[O2] |

**Table ST2:** MM-GBSA residue decomposition results for the selected proteins

| Proteins |  |  |  |  |  |  |  |  |  |
| --- | --- | --- | --- | --- | --- | --- | --- | --- | --- |
|  |  |  |  |  |  |  |  |  |  |
| 100 ns | Dg bind | Coulomb | Solvation | Covalent | Vdw | H-bond | Lipo | Pi-Pi | Contact |
|  |  |  |  |  |  |  |  |  |  |
| **CLUH** | **-30.31** | **-6.2** | **7.92** | **0** | **-24.04** | **-0.44** | **-8.43** | **0** | **0** |
| GLUD1 | -16.35 | -2.31 | 8.9 | 0 | -18.58 | -0.32 | -5.64 | 0 | 0 |
| **CYP51A1** | **-30.69** | **-4.42** | **9.96** | **0** | **-24.58** | **-0.38** | **-13.02** | **0** | **0** |
| LSS | -15.48 | -2.16 | 3.93 | 0 | -12.46 | -0.11 | -5.77 | 0 | 0 |
| **P4HB** | **-30.02** | **-8.68** | **8.89** | **0** | **-23.36** | **-0.54** | **-8.85** | **0** | **0** |
| PITRM1 | -14.23 | -2.44 | 6.56 | 0 | -12.91 | -0.11 | -6.36 | 0 | 0 |

**Table ST3:** Tunnels and channels detected in CLUH, CYP51A1, LSS, GLUD1, P4HB and PITRM1

**Tunnel**-identifier of protein tunnel; **Bottleneck radius**-radius of the narrowest part of the tunnel; **Length**-Length of the tunnel; **Curvature**-curvature of the tunnel; **Throughput**-throughput of the tunnel

| **Details of Tunnel** | **S. No** |  |  |  |  |  |  |  |  |  |
| --- | --- | --- | --- | --- | --- | --- | --- | --- | --- | --- |
| **GLUD1** | **Tunnel** | **Bottleneck Radius [A˚]:** | **Length**  **[A˚]:** | **Distance to surface**  **[A˚]:** | **Distance from starting point [A˚]:** | **Curvature** | **Throughput** | **Number of Residues** | **Number of Bottleneck** | **Bottleneck Residues (12)** |
|  | 2 | 1.8 | 9.2 | 7.9 | 0.5 | 1.2 | 0.77 | 23 | 1 | **ALA223, PRO224, ASP225, GLY229,**  **GLY257,**  **ARG268, ILE269, THR272, ASN311, VAL312, GLY434, THR228** |
|  |  |  |  |  |  |  |  |  |  |  |
| **CYP51A1** | **Tunnel** | **Bottleneck Radius [A˚]:** | **Length**  **[A˚]:** | **Distance to surface**  **[A˚]:** | **Distance from starting point [A˚]:** | **Curvature** | **Throughput** | **Number of Residues** | **Number of Bottleneck** | **Bottleneck Residues (7)** |
|  | 1 | 1.7 | 9.7 | 8.4 | 7.5 | 1.2 | 0.79 | 22 | 1 | **ASP231, PHE234, THR235, HIS314, THR486, MET487, ILE488** |
|  |  |  |  |  |  |  |  |  |  |  |
|  |  |  |  |  |  |  |  |  |  | **Bottleneck Residues (9)** |
|  | 2 | 2.0 | 15.9 | 12.7 | 4.5 | 1.3 | 0.76 | 33 | 1 | **TYR131, LEU134, ILE377, MET378, THR379, MET380, MET381, MET487, ILE488** |
|  |  |  |  |  |  |  |  |  |  |  |
|  | **Tunnel** | **Bottleneck Radius [A˚]:** | **Length**  **[A˚]:** | **Distance to surface**  **[A˚]:** | **Distance from staring point [A˚]:** | **Curvature** | **Throughput** | **Number of Residues** | **Number of Bottleneck** | **Bottleneck Residues (7)** |
| **CLUH** | 6 | 1.0 | 24.1 | 12.4 | 8.5 | 1.9 | 0.45 | 34 | 1 | **LYS140, ASP143, PRO144, SER145, ASP146, ALA147, ASN154** |
|  |  |  |  |  |  |  |  |  |  |  |
| **PITRM1**  **(No bottleneck amino acid reported for substrate binding)** | **Tunnel** | **Bottleneck Radius [A˚]:** | **Length**  **[A˚]:** | **Distance to surface**  **[A˚]:** | **Distance from staring point [A˚]:** | **Curvature** | **Throughput** | **Number of Residues** | **Number of Bottleneck** | **Bottleneck Residues (10)** |
|  | 1 | 1.6 | 51.6 | 28.4 | 0.0 | 1.8 | 0.67 | 76 | 1 | **HIS104, GLU107, HIS108, ASN136, ALA137, MET138, THR139, VAL202, GLU205, TYR905** |
|  |  |  |  |  |  |  |  |  |  |  |
| **LSS**  **(No bottleneck amino acid reported for substrate binding)** | **Tunnel** | **Bottleneck Radius [A˚]:** | **Length**  **[A˚]:** | **Distance to surface**  **[A˚]:** | **Distance from staring point [A˚]:** | **Curvature** | **Throughput** | **Number of Residues** | **Number of Bottleneck** | **Bottleneck Residues (11)** |
|  | 1 | 1.0 | 17.1 | 13.9 | 11.0 | 1.2 | 0.44 | 33 | 1 | **TRP388, PHE392, GLN395, ASP456, GLU460, LYS463, ALA537, GLN540, TRP591, PHE592, TRP715** |
|  |  |  |  |  |  |  |  |  |  |  |
|  | **Tunnel** | **Bottleneck Radius [A˚]:** | **Length**  **[A˚]:** | **Distance to surface**  **[A˚]:** | **Distance from staring point [A˚]:** | **Curvature** | **Throughput** | **Number of Residues** | **Number of Bottleneck** | **Bottleneck Residues (8)** |
|  | 2 | 0.9 | 26.2 | 15.6 | 8.0 | 1.7 | 0.33 | 36 | 1 | **TRP231, CYS232, HIS233, THR503, TYR504, ASP528, TYR531, TRP582** |
|  |  |  |  |  |  |  |  |  |  |  |
| **P4HB**  **(No bottleneck amino acid reported for substrate binding)** | **Tunnel** | **Bottleneck Radius [A˚]:** | **Length**  **[A˚]:** | **Distance to surface**  **[A˚]:** | **Distance from staring point [A˚]:** | **Curvature** | **Throughput** | **Number of Residues** | **Number of Bottleneck** | **Bottleneck Residues (7)** |
|  | 22 | 1.0 | 42.8 | 30.9 | 23.0 | 1.4 | 0.30 | 42 | 1 | **ILE250, PHE251, GLY252, LYS256, HIS258, LEU322, MET326** |

**Table ST4:** Atoms positioning of amino acids inside the tunnel during bond formation with the substrate for GLUD1, CLUH, CYP51A1

| **CYP51A1** | **S No:** | **ID** | **Name** |
| --- | --- | --- | --- |
|  | Tunnel |  |  |
| **ASP231** | 1 | 3596 | CA |
|  |  | 3597 | C |
|  |  | 3598 | CB |
|  |  | 3599 | O |
|  |  | 3600 | CG |
|  |  | 3601 | OD1 |
|  |  | 3602 | OD2 |
|  |  | 3604 | HA |
|  |  | 3605 | HB2 |
|  |  | 3606 | HB3 |
|  |  |  |  |
| **PHE234** | 1 | 3621 | N |
|  |  | 3622 | CA |
|  |  | 3623 | C |
|  |  | 3624 | CB |
|  |  | 3625 | O |
|  |  | 3626 | CG |
|  |  | 3627 | CD1 |
|  |  | 3628 | CD2 |
|  |  | 3629 | CE1 |
|  |  | 3630 | CE2 |
|  |  | 3631 | CZ |
|  |  | 3632 | H |
|  |  | 3633 | HA |
|  |  | 3634 | HB2 |
|  |  | 3635 | HB3 |
|  |  | 3636 | HD1 |
|  |  | 3637 | HD2 |
|  |  | 3638 | HE1 |
|  |  | 3639 | HE2 |
|  |  | 3640 | HZ |
|  |  |  |  |
| **MET487** | 1 | 7729 | N |
|  |  | 7730 | CA |
|  |  | 7731 | C |
|  |  | 7732 | CB |
|  |  | 7733 | O |
|  |  | 7734 | CG |
|  |  | 7735 | SD |
|  |  | 7736 | CE |
|  |  | 7737 | H |
|  |  | 7738 | HA |
|  |  | 7739 | HB2 |
|  |  | 7740 | HB3 |
|  |  | 7741 | HG2 |
|  |  | 7742 | HG3 |
|  |  | 7743 | HE1 |
|  |  | 7744 | HE2 |
|  |  | 7745 | HE3 |
|  |  |  |  |
|  |  |  |  |
| **ILE488** | 1 | 7746 | N |
|  |  | 7747 | CA |
|  |  | 7748 | C |
|  |  | 7749 | CB |
|  |  | 7750 | O |
|  |  | 7751 | CG1 |
|  |  | 7752 | CG2 |
|  |  | 7753 | CD1 |
|  |  | 7754 | H |
|  |  | 7755 | HA |
|  |  | 7756 | HB |
|  |  | 7757 | HG12 |
|  |  | 7758 | HG13 |
|  |  | 7761 | HD23 |
|  |  | 7762 | HD11 |
|  |  | 7763 | HD12 |
|  |  | 7764 | HD13 |
|  |  |  |  |
|  |  |  |  |
| **TYR131** | 2 | 1984 | CA |
|  |  | 1985 | C |
|  |  | 1986 | CB |
|  |  | 1987 | O |
|  |  | 1988 | CG |
|  |  | 1989 | CD1 |
|  |  | 1990 | CD2 |
|  |  | 1991 | CE1 |
|  |  | 1992 | CE2 |
|  |  | 1993 | OH |
|  |  | 1994 | CZ |
|  |  | 1996 | HA |
|  |  | 1997 | HB2 |
|  |  | 1998 | HB3 |
|  |  | 1999 | HD1 |
|  |  | 2000 | HD2 |
|  |  | 2001 | HE1 |
|  |  | 2002 | HE2 |
|  |  | 2003 | HH |
|  |  |  |  |
| **ILE377** | 2 | 5976 | N |
|  |  | 5977 | CA |
|  |  | 5978 | C |
|  |  | 5979 | CB |
|  |  | 5980 | O |
|  |  | 5981 | CG1 |
|  |  | 5982 | CG2 |
|  |  | 5983 | CD1 |
|  |  | 5985 | HA |
|  |  | 5986 | HB |
|  |  | 5987 | HG12 |
|  |  | 5988 | HG13 |
|  |  | 5989 | HG21 |
|  |  | 5990 | HG22 |
|  |  | 5991 | HG23 |
|  |  | 5992 | HD11 |
|  |  | 5993 | HD12 |
|  |  | 5994 | HD13 |
|  |  |  |  |
| **MET487** | 2 | 7729 | N |
|  |  | 7730 | CA |
|  |  | 7731 | C |
|  |  | 7732 | CB |
|  |  | 7733 | O |
|  |  | 7734 | CG |
|  |  | 7735 | SD |
|  |  | 7736 | CE |
|  |  | 7737 | H |
|  |  | 7738 | HA |
|  |  | 7739 | HB2 |
|  |  | 7740 | HB3 |
|  |  | 7741 | HG2 |
|  |  | 7742 | HG3 |
|  |  | 7743 | HE1 |
|  |  | 7744 | HE2 |
|  |  | 7745 | HE3 |
|  |  |  |  |
|  |  |  |  |
| **ILE488** | 2 | 7746 | N |
|  |  | 7747 | CA |
|  |  | 7748 | C |
|  |  | 7749 | CB |
|  |  | 7750 | O |
|  |  | 7751 | CG1 |
|  |  | 7752 | CG2 |
|  |  | 7753 | CD1 |
|  |  | 7754 | H |
|  |  | 7755 | HA |
|  |  | 7756 | HB |
|  |  | 7757 | HG12 |
|  |  | 7758 | HG13 |
|  |  | 7761 | HD23 |
|  |  | 7762 | HD11 |
|  |  | 7763 | HD12 |
|  |  | 7764 | HD13 |
|  |  |  |  |
| **GLUD1** |  |  |  |
| **ARG268** | 2 | 4087 | CA |
|  |  | 4088 | C |
|  |  | 4089 | CB |
|  |  | 4090 | O |
|  |  | 4091 | CG |
|  |  | 4092 | CD |
|  |  | 4093 | NE |
|  |  | 4094 | NH1 |
|  |  | 4095 | NH2 |
|  |  | 4096 | CZ |
|  |  | 4101 | HG2 |
|  |  | 4102 | HG3 |
|  |  | 4104 | HD3 |
|  |  | 4105 | HE |
|  |  | 4106 | HH11 |
|  |  | 4107 | HH12 |
|  |  | 4108 | HH21 |
|  |  | 4109 | HH22 |
|  |  |  |  |
| **THR272** | 2 | 4150 | N |
|  |  | 4151 | CA |
|  |  | 4153 | CB |
|  |  | 4155 | CG2 |
|  |  | 4156 | OG1 |
|  |  | 4157 | H |
|  |  | 4159 | HB |
|  |  | 4160 | HG21 |
|  |  | 4161 | HG22 |
|  |  | 4162 | HG23 |
|  |  | 4163 | HG1 |
|  |  |  |  |
| **ASN311** | 2 | 4723 | N |
|  |  | 4724 | CA |
|  |  | 4725 | C |
|  |  | 4726 | CB |
|  |  | 4727 | O |
|  |  | 4728 | CG |
|  |  | 4729 | ND2 |
|  |  | 4730 | OD1 |
|  |  | 4731 | H |
|  |  | 4732 | HA |
|  |  | 4733 | HB2 |
|  |  | 4734 | HB3 |
|  |  | 4735 | HD21 |
|  |  | 4736 | HD22 |
|  |  |  |  |
| **CLUH** |  |  |  |
| **ASP143** | 6 | 2210 | N |
|  |  | 2211 | CA |
|  |  | 2212 | C |
|  |  | 2213 | CB |
|  |  | 2214 | O |
|  |  | 2215 | CG |
|  |  | 2216 | OD1 |
|  |  | 2217 | OD2 |
|  |  | 2218 | H |
|  |  | 2219 | HA |
|  |  | 2220 | HB2 |
|  |  | 2221 | HB3 |

**Table ST5:** Alanine Scanning of binding amino acids in Abiraterone complex

| **3LD6_Abiraterone_**  **Alanine_Scanning** | **Mutation** | **Affinity**  (kcal/mol) | **dAffinity**  (kcal/mol) | **Stability**  (kcal/mol) | **dStability**  (kcal/mol) |
| --- | --- | --- | --- | --- | --- |
|  |  |  |  |  |  |
|  | **F77F** | -267.4190 | 0.0000 | -1449.5120 | 0.0000 |
|  | **F77A** | -257.3097 | 10.1094 | -1447.0289 | 2.4831 |
|  |  |  |  |  |  |
|  | **F105F** | -7.4065 | 0.0000 | -1448.9657 | 0.0000 |
|  | **F105A** | -7.1888 | 0.2177 | -1446.0617 | 2.9040 |
|  |  |  |  |  |  |
|  | **Y131Y** | -258.3662 | 0.0000 | -1446.2978 | 0.0000 |
|  | **Y131A** | -253.7609 | 4.6053 | -1444.1115 | 2.1863 |
|  |  |  |  |  |  |
|  | **L134L** | -262.4248 | 0.0000 | -1447.0563 | 0.0000 |
|  | **L134A** | -259.3465 | 3.0783 | -1444.6060 | 2.4502 |
|  |  |  |  |  |  |
|  | **T135T** | -247.7128 | 0.0000 | -1444.6931 | 0.0000 |
|  | **T135A** | -258.8932 | -11.1804 | -1444.5308 | 0.1623 |
|  |  |  |  |  |  |
|  | **F234F** | -264.0480 | 0.0000 | -1447.9761 | 0.0000 |
|  | **F234A** | -259.0611 | 4.9869 | -1445.6090 | 2.3671 |
|  |  |  |  |  |  |
|  | **H236H** | -263.9910 | 0.0000 | -1445.2467 | 0.0000 |
|  | **H236A** | -259.4069 | 4.5841 | -1444.3741 | 0.8726 |
|  |  |  |  |  |  |
|  | **W239W** | -261.4303 | 0.0000 | -1448.2971 | 0.0000 |
|  | **W239A** | -254.7819 | 6.6484 | -1445.2002 | 3.0969 |
|  |  |  |  |  |  |
|  | **I377I** | -264.5705 | 0.0000 | -1447.5105 | 0.0000 |
|  | **I377A** | -261.6117 | 2.9588 | -1445.9660 | 1.5444 |
|  |  |  |  |  |  |
|  | **I379I** | -265.8310 | 0.0000 | -1448.4057 | 0.0000 |
|  | **I379A** | -261.8254 | 4.0056 | -1446.1816 | 2.2241 |
|  |  |  |  |  |  |
|  | **M381M** | -261.9309 | 0.0000 | -1448.3179 | 0.0000 |
|  | **M381A** | -256.4915 | 5.4394 | -1446.3762 | 1.9417 |
|  |  |  |  |  |  |
|  | **M487M** | -265.3547 | 0.0000 | -1446.3182 | 0.0000 |
|  | **M487A** | -264.1815 | 1.1733 | -1445.6865 | 0.6317 |

**Table ST6:** Alanine Scanning of binding amino acids of Pregnenolone(P5) complex in Abiraterone binding

pocket

| **3LD6_Pregnenolone(P5)**  **_Alanine_Scanning** | **Mutation** | **Affinity**  (kcal/mol) | **dAffinity**  (kcal/mol) | **Stability**  (kcal/mol) | **dStability**  (kcal/mol) |
| --- | --- | --- | --- | --- | --- |
|  |  |  |  |  |  |
|  | **Y131Y** | -7.4053 | 0.0000 | -1442.7802 | 0.0000 |
|  | **Y131A** | -7.1000 | 0.3053 | -1440.8535 | 1.9266 |
|  |  |  |  |  |  |
|  | **L134L** | -7.2518 | 0.0000 | -1443.7848 | 0.0000 |
|  | **L134A** | -7.1089 | 0.1428 | -1441.4081 | 2.3767 |
|  |  |  |  |  |  |
|  | **V138V** | -7.2128 | 0.0000 | -1445.7197 | 0.0000 |
|  | **V138A** | -7.1953 | 0..0175 | -1444.3547 | 1.3649 |
|  |  |  |  |  |  |
|  | **F139F** | -7.2066 | 0.0000 | -1444.8488 | 0.0000 |
|  | **F139A** | -7.2507 | -0.0441 | -1442.7531 | 2.0956 |
|  |  |  |  |  |  |
|  | **K156K** | -7.2268 | 0.0000 | -1441.8246 | 0.0000 |
|  | **K156A** | -7.2245 | 0.0022 | -1440.5688 | 1.2558 |
|  |  |  |  |  |  |
|  | **F234F** | -7.2659 | 0.0000 | -1445.1901 | 0.0000 |
|  | **F234A** | -7.2388 | 0.0271 | -1442.5044 | 2.6857 |
|  |  |  |  |  |  |
|  | **A311A** | -7.2235 | 0.0000 | -1443.2380 | 0.0000 |
|  |  |  |  |  |  |
|  | **I377I** | -7.2671 | 0.0000 | -1444.1849 | 0.0000 |
|  | **I377A** | -7.1546 | 0.1125 | -1442.5279 | 1.6570 |
|  |  |  |  |  |  |
|  | **I379I** | -7.2234 | 0.0000 | -1445.4187 | 0.0000 |
|  | **I379A** | -7.0054 | 0.2180 | -1443.0346 | 2.3842 |
|  |  |  |  |  |  |
|  | **M380M** | -7.2377 | 0.0000 | -1444.5244 | 0.0000 |
|  | **M380A** | -7.3181 | -0.0804 | -1442.8022 | 1.7222 |
|  |  |  |  |  |  |
|  | **M487M** | -7.2043 | 0.0000 | -1443.5727 | 0.0000 |
|  | **M487A** | -6.8663 | 0.3380 | -1442.1486 | 1.4241 |

**Table ST7:** Alanine Scanning of binding amino acids of lanosterol complex

| **6UEZ_Lanosterol**  **_Alanine_Scanning** | **Mutation** | **Affinity**  (kcal/mol) | **dAffinity**  (kcal/mol) | **Stability**  (kcal/mol) | **dStability**  (kcal/mol) |
| --- | --- | --- | --- | --- | --- |
|  |  |  |  |  |  |
|  | **Y131Y** | -49.2360 | 0.0000 | 4598.7933 | 0.0000 |
|  | **Y131A** | -48.1617 | 1.0743 | 4601.7140 | 2.9207 |
|  |  |  |  |  |  |
|  | **L134L** | -49.3350 | 0.0000 | 4598.2688 | 0.0000 |
|  | **L134A** | -49.1179 | 0.2171 | 4600.6431 | 2.3743 |
|  |  |  |  |  |  |
|  | **A144A** | -49.2827 | 0.0000 | 4597.9627 | 0.0000 |
|  |  |  |  |  |  |
|  | **Y145Y** | -48.6624 | 0.0000 | 4598.8450 | 0.0000 |
|  | **Y145A** | -47.4999 | 1.1625 | 4601.5844 | 2.7394 |
|  |  |  |  |  |  |
|  | **F152F** | -49.3830 | 0.0000 | 4597.1299 | 0.0000 |
|  | **F152A** | -48.7221 | 0.6609 | 4599.4119 | 2.2820 |
|  |  |  |  |  |  |
|  | **L159L** | -49.3397 | 0.0000 | 4597.7900 | 0.0000 |
|  | **L159A** | -49.0556 | 0.2841 | 4600.2600 | 2.4699 |
|  |  |  |  |  |  |
|  | **F234F** | -49.2484 | 0.0000 | 4596.8603 | 0.0000 |
|  | **F234A** | -48.7683 | 0.4801 | 4599.6463 | 2.7861 |
|  |  |  |  |  |  |
|  | **M304M** | -49.3411 | 0.0000 | 4592.3215 | 0.0000 |
|  | **M304A** | -49.2929 | 0.0481 | 4594.7662 | 2.4448 |
|  |  |  |  |  |  |
|  | **L310L** | -49.1245 | 0.0000 | 4595.9581 | 0.0000 |
|  | **L310A** | -49.0454 | 0.0792 | 4598.1881 | 2.2300 |
|  |  |  |  |  |  |
|  | **A311A** | -49.2681 | 0.0000 | 4559.0609 | 0.0000 |
|  |  |  |  |  |  |
|  | **I377I** | -48.9984 | 0.0000 | 4598.2592 | 0.0000 |
|  | **I377A** | -48.1135 | 0.8849 | 4600.3433 | 2.0842 |
|  |  |  |  |  |  |
|  | **I379I** | -48.8909 | 0.0000 | 4597.1562 | 0.0000 |
|  | **I379A** | -48.8061 | 0.0847 | 4599.7319 | 2.5757 |
|  |  |  |  |  |  |
|  | **M487M** | -49.3235 | 0.0000 | 4597.5711 | 0.0000 |
|  | **M487A** | -49.1813 | 0.1422 | 4599.6765 | 2.1054 |
|  |  |  |  |  |  |
|  | **I488I** | -49.3079 | 0.000 | 4596.9460 | 0.0000 |
|  | **I488A** | -48.9485 | 0.3594 | 4599.5018 | 2.5558 |

**Table ST8:** Alanine Scanning of binding amino acids of pregnenolone(P5) complex in Lanosterol binding pocket

| **6UEZ_Pregnenolone(P5)**  **_Alanine_Scanning** | **Mutation** | **Affinity**  (kcal/mol) | **dAffinity**  (kcal/mol) | **Stability**  (kcal/mol) | **dStability**  (kcal/mol) |
| --- | --- | --- | --- | --- | --- |
|  |  |  |  |  |  |
|  | **Y131Y** | -7.4063 | 0.0000 | -1442.7631 | 0.0000 |
|  | **Y131A** | -7.0963 | 0.3099 | -1440.8480 | 1.9151 |
|  |  |  |  |  |  |
|  | **L134L** | -7.2042 | 0.0000 | -1443.8642 | 0.0000 |
|  | **L134A** | -7.1876 | 0.0167 | 1441.4669 | 2.3974 |
|  |  |  |  |  |  |
|  | **F139F** | -7.2083 | 0.0000 | -1444.8521 | 0.0000 |
|  | **F139A** | -7.2542 | -0.0459 | -1442.7579 | 2.0941 |
|  |  |  |  |  |  |
|  | **A144A** | -7.2279 | 0.0000 | -1443.9531 | 0.0000 |
|  |  |  |  |  |  |
|  | **F152F** | -7.2279 | 0.0000 | -1444.8095 | 0.0000 |
|  | **F152A** | -7.2270 | 0.0009 | -1442.8471 | 1.9625 |
|  |  |  |  |  |  |
|  | **F234F** | -7.2678 | 0.0000 | -1445.1750 | 0.0000 |
|  | **F234A** | -7.2377 | 0.0301 | -1442.5252 | 2.6498 |
|  |  |  |  |  |  |
|  | **L310L** | -7.2344 | 0.0000 | -1443.5028 | 0.0000 |
|  | **L310A** | -7.2305 | 0.0040 | -1441.3323 | 2.1705 |
|  |  |  |  |  |  |
|  | **A311A** | -7.2248 | 0.0000 | -1443.2248 | 0.0000 |
|  |  |  |  |  |  |
|  | **H314H** | -7.2523 | 0.0000 | -1442.3339 | 0.0000 |
|  | **H314A** | -7.2356 | 0.0167 | -1441.4410 | 0.8929 |
|  |  |  |  |  |  |
|  | **I377I** | -7.2606 | 0.0000 | -1444.1750 | 0.0000 |
|  | **I377A** | -7.1345 | 0.1261 | -1442.5413 | 1.6337 |
|  |  |  |  |  |  |
|  | **I379I** | -7.2315 | 0.0000 | -1445.3568 | 0.0000 |
|  | **I379A** | -7.0206 | 0.2108 | -1443.0307 | 2.3261 |
|  |  |  |  |  |  |
|  | **I450I** | -7.2259 | 0.0000 | -1444.5457 | 0.0000 |
|  | **I450A** | -7.2259 | 0.0000 | -1442.2728 | 2.2729 |
|  |  |  |  |  |  |
|  | **I488I** | -7.2048 | 0.0000 | -1444.7417 | 0.0000 |
|  | **I488A** | -7.1668 | 0.0380 | -1442.4099 | 2.3317 |

**Table ST9:** Alanine Scanning of pregnenolone(P5) complex with CLUH, P4HB and CYP51A1 modeled proteins

| **CLUH_AlphaFold_**  **Pregnenolone(P5) _**  **Alanine_Scanning** | **Mutation** | **Affinity**  (kcal/mol) | **dAffinity**  (kcal/mol) | **Stability**  (kcal/mol) | **dStability**  (kcal/mol) |
| --- | --- | --- | --- | --- | --- |
|  |  |  |  |  |  |
|  | **R136R** | **-7.2383** | **0.0000** | **-4669.3280** | **0.0000** |
|  | **R136A** | **-7.1996** | **0.0387** | **-4667.4721** | **1.8559** |
|  |  |  |  |  |  |
|  | **L139L** | **-7.3432** | **0.0000** | **-4680.9993** | **0.0000** |
|  | **L139A** | **-7.1207** | **0.2226** | **-4678.5714** | **2.4279** |
|  |  |  |  |  |  |
|  | **K140K** | **-7.0100** | **0.0000** | **-4678.2785** | **0.0000** |
|  | **K140A** | **-6.9811** | **0.0289** | **-4676.7089** | **1.5697** |
|  |  |  |  |  |  |
|  | **D143D** | **-7.3862** | **0.0000** | **-4675.4775** | **0.0000** |
|  | **D143A** | **-7.0879** | **0.2983** | **-4674.8929** | **0.5846** |
|  |  |  |  |  |  |
|  | **P211P** | **-7.7076** | **0.0000** | **-4682.2138** | **0.0000** |
|  | **P211A** | **-7.6621** | **0.0454** | **-4682.1756** | **0.0382** |
|  |  |  |  |  |  |
|  | **L212L** | **-7.4798** | **0.0000** | **-4681.0899** | **0.0000** |
|  | **L212A** | **-7.4305** | **0.0493** | **-4678.9095** | **2.1805** |
|  |  |  |  |  |  |
|  | **L218L** | **-7.5859** | **0.0000** | **-4681.4778** | **0.0000** |
|  | **L218A** | **-7.5419** | **0.0440** | **-4679.0346** | **2.4431** |
|  |  |  |  |  |  |
|  | **D332D** | **-7.1788** | **0.0000** | **-4675.1707** | **0.0000** |
|  | **D332A** | **-7.0363** | **0.1425** | **-4674.3359** | **0.8348** |
|  |  |  |  |  |  |
| **P4HB_AlphaFold_**  **Pregnenolone(P5) _**  **Alanine_Scanning** | **Mutation** | **Affinity**  (kcal/mol) | **dAffinity**  (kcal/mol) | **Stability**  (kcal/mol) | **dStability**  (kcal/mol) |
|  |  |  |  |  |  |
|  | **K232K** | **-7.2063** | **0.0000** | **-1620.1055** | **0.0000** |
|  | **K232A** | **-6.8587** | **0.3476** | **-1619.2302** | **0.8754** |
|  |  |  |  |  |  |
|  | **H233H** | **-7.1384** | **0.0000** | **-1619.6670** | **0.0000** |
|  | **H233A** | **-7.0950** | **0.0433** | **-1619.2245** | **0.4425** |
|  |  |  |  |  |  |
|  | **Q235Q** | **-7.4369** | **0.0000** | **-1617.6262** | **0.0000** |
|  | **Q235A** | **-7.4034** | **0.0335** | **-1617.1580** | **0.4681** |
|  |  |  |  |  |  |
|  | **L236L** | **-7.7020** | **0.0000** | **-1622.7901** | **0.0000** |
|  | **L236A** | **-7.5585** | **0.1435** | **-1620.5462** | **2.2439** |
|  |  |  |  |  |  |
|  | **L238L** | **-7.3855** | **0.0000** | **-1622.5447** | **0.0000** |
|  | **L238A** | **-7.3000** | **0.0855** | **-1620.9925** | **1.5222** |
|  |  |  |  |  |  |
|  | **I240I** | **-7.2143** | **0.0000** | **-1624.4228** | **0.0000** |
|  | **I240A** | **-7.1904** | **0.0239** | **-1622.0559** | **2.3669** |
|  |  |  |  |  |  |
|  | **K249K** | **-7.6353** | **0.0000** | **-1620.8084** | **0.0000** |
|  | **K249A** | **-7.2739** | **0.3614** | **-1619.6748** | **1.1336** |
|  |  |  |  |  |  |
|  | **I250I** | **-7.2833** | **0.0000** | **-1624.5214** | **0.0000** |
|  | **I250A** | **-7.2434** | **0.0399** | **-1621.9481** | **2.5733** |
|  |  |  |  |  |  |
|  | **K285K** | **-7.3609** | **0.0000** | **-1619.1264** | **0.0000** |
|  | **K285A** | **-7.0962** | **0.2647** | **-1618.6664** | **0.4600** |
|  |  |  |  |  |  |
|  | **G286G** | **-7.2883** | **0.0000** | **-1621.6470** | **0.0000** |
|  | **G286A** | **-7.3712** | **-0.0829** | **-1621.2972** | **0.3498** |
|  |  |  |  |  |  |
|  | **I288I** | **-7.1802** | **0.0000** | **-1624.5509** | **0.0000** |
|  | **I288A** | **-7.1724** | **0.0078** | **-1621.9204** | **2.6305** |
|  |  |  |  |  |  |
|  | **L289L** | **-7.1761** | **0.0000** | **-1622.9923** | **0.0000** |
|  | **L289A** | **-6.8564** | **0.3198** | **-1620.5840** | **2.4083** |
|  |  |  |  |  |  |
|  | **F290F** | **-7.1987** | **0.0000** | **-1624.6630** | **0.0000** |
|  | **F290A** | **-7.1906** | **0.0082** | **-1621.9031** | **2.7599** |
|  |  |  |  |  |  |
|  |  |  |  |  |  |
| **CYP51A1_AlphaFold_**  **Pregnenolone(P5) _**  **Alanine_Scanning** | **Mutation** | **Affinity**  (kcal/mol) | **dAffinity**  (kcal/mol) | **Stability**  (kcal/mol) | **dStability**  (kcal/mol) |
|  |  |  |  |  |  |
|  | **Y131Y** | **-6.7856** | **0.0000** | **-1646.1547** | **0.0000** |
|  | **Y131A** | **-6.1508** | **0.6078** | **-1643.6961** | **2.4586** |
|  |  |  |  |  |  |
|  | **H314H** | **-6.8357** | **0.0000** | **-1645.9434** | **0.0000** |
|  | **H314A** | **-6.8197** | **0.0159** | **-1645.2885** | **0.6550** |
|  |  |  |  |  |  |
|  | **F234F** | **-6.7991** | **0.0000** | **-1649.0601** | **0.0000** |
|  | **F234A** | **-6.4346** | **0.3646** | **-1646.3991** | **2.6610** |
|  |  |  |  |  |  |
|  | **I377I** | **-6.9641** | **0.0000** | **-1648.1073** | **0.0000** |
|  | **I377A** | **-6.4134** | **0.5507** | **-1646.0731** | **2.0341** |
|  |  |  |  |  |  |
|  | **M380M** | **-6.7895** | **0.0000** | **-1647.3333** | **0.0000** |
|  | **M380A** | **-6.4826** | **0.3069** | **-1645.2203** | **2.1130** |
|  |  |  |  |  |  |
|  | **R382R** | **-6.7047** | **0.0000** | **-1634.8391** | **0.0000** |
|  | **R382A** | **-6.6881** | **0.0166** | **-1632.5212** | **2.3179** |
|  |  |  |  |  |  |
|  | **H447H** | **-6.6904** | **0.0000** | **-1645.4561** | **0.0000** |
|  | **H447A** | **-6.9743** | **-0.2839** | **-1644.7310** | **0.7251** |
|  |  |  |  |  |  |
|  | **C449C** | **-6.9154** | **0.0000** | **-1646.4479** | **0.0000** |
|  | **C449A** | **-6.9233** | **-0.0079** | **-1644.7326** | **1.7154** |
